# Seed Yield and Nitrogen Efficiency in Oilseed Rape After Ammonium Nitrate or Urea Fertilization

**DOI:** 10.3389/fpls.2020.608785

**Published:** 2021-01-27

**Authors:** Diana Heuermann, Heike Hahn, Nicolaus von Wirén

**Affiliations:** ^1^Molecular Plant Nutrition, Leibniz Institute of Plant Genetics and Crop Plant Research, Gatersleben, Germany; ^2^Agricultural Application Research, SKW Stickstoffwerke Piesteritz GmbH, Borsdorf, Germany

**Keywords:** nitrogen uptake, ammonium nitrate, urea, cytokinin translocation, nitrogen use efficiency, nitrogen uptake efficiency, nitrogen partitioning, rapeseed

## Abstract

In agricultural plant production, nitrate, ammonium, and urea are the major fertilized nitrogen forms, which differ in root uptake and downstream signaling processes in plants. Nitrate is known to stimulate cytokinin synthesis in roots, while for urea no hormonal effect has been described yet. Elevated cytokinin levels can delay plant senescence favoring prolonged nitrogen uptake. As the cultivation of winter oilseed rape provokes high nitrogen-balance surpluses, we tested the hypotheses whether nitrogen use efficiency increases under ammonium nitrate- relative to urea-based nutrition and whether this is subject to genotypic variation. In a 2-year field study, 15 oilseed rape lines were fertilized either with ammonium nitrate or with urease inhibitor-stabilized urea and analyzed for seed yield and nitrogen-related yield parameters. Despite a significant environmental impact on the performance of the individual lines, which did not allow revealing consistent impact of the genotype, ammonium nitrate-based nutrition tended to increase seed yield in average over all lines. To resolve whether the fertilizer N forms act on grain yield via phytohormones, we collected xylem exudates at three developmental stages and determined the translocation rates of cytokinins and N forms. Relative to urea, ammonium nitrate-based nutrition enhanced the translocation of nitrate or total nitrogen together with cytokinins, whereas in the urea treatment translocation rates were lower as long as urea remained stable in the soil solution. At later developmental stages, i.e., when urea became hydrolyzed, nitrogen and cytokinin translocation increased. In consequence, urea tended to increase nitrogen partitioning in the shoot toward generative organs. However, differences in overall nitrogen accumulation in shoots were not present at the end of the vegetation period, and neither nitrogen uptake nor utilization efficiency was consistently different between the two applied nitrogen forms.

## Introduction

In Northern Europe, winter oilseed rape is nowadays grown as the most important oil crop (Rathke et al., 2006). Relative to other crops, agricultural production of rape causes high surpluses in the nitrogen (N) balance (Schulte auf’m Erley et al., 2011), which is a major source for N losses to the environment and represents an important problem in sustainable crop production. Thus, it is of increasing importance in oilseed rape production to reduce the amount of applied N fertilizer as well as of N-rich residues remaining on the field after harvest (Schulte auf’m Erley et al., 2011).

The capacity of a crop to convert soil-available N into grain yield is described by its N use efficiency (NUE). NUE depends on the efficiency for N uptake (NupE) as well as on the efficiency for utilizing absorbed N for grain production (NutE, Moll et al., 1982). Oilseed rape is generally characterized by a high capacity for N uptake during vegetative growth, while after flowering N uptake decreases drastically (Malagoli et al., 2004). Hence, N required for seed filling derives from remobilization from vegetative organs (Rossato et al., 2001), but due to insufficient sink strength N transfer to the seeds remains low (Sveènjak and Rengel, 2006). In order to improve NUE of oilseed rape, it has been suggested to focus breeding efforts on N uptake during generative plant development and on N retranslocation from vegetative organs to seeds (Wiesler et al., 2001a). Regarding these two traits, however, the N supply level plays an important role (Bouchet et al., 2016). For instance, NutE has been repeatedly reported to be more important for determining NUE at high N levels (Berry et al., 2010; Schulte auf’m Erley et al., 2011; Kessel et al., 2012). With regard to the need for reducing fertilizer inputs, the risk for N limitation during oilseed rape cultivation increases. Under these circumstances, tackling NupE appears to be more relevant to improve NUE. To date, several studies on NUE in oilseed rape focused on genotypic differences in NupE and NutE at different N levels (e.g., Schulte auf’m Erley et al., 2011; Kessel et al., 2012; Nyikako et al., 2014; Stahl et al., 2015), while the impact of N forms on NUE has been poorly studied, although N forms may affect N uptake and N partitioning in winter oilseed rape as well. This research question has so far been rarely addressed, and if so with inconsistent results (Fismes et al., 2000; Arkoun et al., 2012).

Most plants are able to take up and assimilate N in the forms of nitrate, ammonium, amino acids (AAs), and urea (Crawford and Glass, 1998). In most soils, fertilized or organic matter-derived urea is rapidly hydrolyzed to ammonium by ubiquitously occurring ureases and concurrently nitrified to nitrate by microorganisms (Mérigout et al., 2008). Root uptake of these different N forms is mainly catalyzed by high-affinity and low-affinity membrane proteins that are specialized for the transport of either N form (Hachiya and Sakakibara, 2016). However, in oilseed rape the uptake rate of urea as sole N form is up to ten times lower than that of nitrate or ammonium (Arkoun et al., 2012). Inside plant cells, nitrate is reduced to ammonium before assimilation, resulting first in glutamine (Gln) and glutamate synthesis (Lam et al., 1996). Absorbed urea is hydrolyzed by cytosolic urease to two molecules of ammonium (Witte, 2011) serving for AA synthesis. When N is limited, nitrate assimilation takes place primarily in roots, while at higher external N concentrations nitrate assimilation in shoots becomes increasingly important (Andrews, 1986). Ammonium, either absorbed or produced in the root, is rather assimilated in roots and ammonium-derived N is then transported to the shoot mainly in the form of AAs (Finnemann and Schjoerring, 1999). Indeed, most plant species transport N in their xylem mainly in the form of amides, such as glutamine and asparagine, and of acidic AAs, such as glutamate and aspartate (Asp) (Fischer et al., 1998). However, also nitrate (Liptay and Arevalo, 2000), ammonium (Finnemann and Schjoerring, 1999), and urea (Liu et al., 2003) are translocated to the shoot. Thus, uptake and assimilation of different N forms change the forms of N delivery from roots to shoots.

The uptake of different N forms can also affect the homeostasis of phytohormones. In particular, cytokinins (CKs) are strongly influenced by the amount and form of N uptake. Together with their ribosides, *N*^6^-isopentenyl-adenine (IP), *trans*-zeatin (Z), *cis*-zeatin (cZ) and dihydrozeatin are the most abundant forms of CKs in higher plants (Sakakibara, 2006) and are mainly synthesized in active root meristems (Engels et al., 2012). The N forms nitrate and ammonium (Takei et al., 2004a), but also Gln (Kamada-Nobusada et al., 2013), stimulate the gene expression of isopentenyltransferases, which catalyze the key step in CK biosynthesis (Takei et al., 2004a; Sakakibara, 2006). Nonetheless, only nitrate-supplied roots translocate in the xylem large amounts of CKs from roots to shoots (Rahayu et al., 2005; Bauer and von Wirén, 2020). In shoots, CKs retard leaf senescence (Jibran et al., 2013) and their exogenous application led to higher seed yield in different species like rice (Ray et al., 1983) and barley (Mishra and Gaur, 1985). Also, the stimulation of higher internal CK levels by downregulating CK oxidases or by naturally lowering the expression of CK-catabolizing enzymes resulted in higher grain number in rice (Ashikari et al., 2005), in larger grain size in wheat (Jameson and Song, 2015), or in improved seed and lint yield in cotton (Zhao et al., 2015). In contrast, urea as N form appears not to be able to delay leaf senescence (Schildhauer et al., 2008).

Based on the above-described mechanisms, we hypothesized that relative to urea ammonium nitrate-based N nutrition of oilseed rape improves NupE in two ways: (i) Ammonium nitrate increases total N uptake due to higher uptake capacities for nitrate and ammonium compared to urea, and (ii) nitrate prolongs the phase of N uptake by stimulating the production of CKs in roots as well as their root-to-shoot translocation via the xylem, leading to delayed leaf senescence and prolonged assimilate supply to roots. This may help to maintain root N uptake activity also during generative plant development. Thus, in a field experiment we examined a set of 15 winter oilseed rape genotypes with differential N efficiency according to previous field experiments by different German breeders. Plants were fertilized with N in the form of ammonium nitrate or urea stabilized by a urease inhibitor to prolong urea availability. Ammonium nitrate was supposed to establish a rather nitrate-based nutrition, as a large part of the ammonium is either rapidly converted to nitrate or adsorbing to the soil matrix whenever the soil’s cation exchange capacity is high (Bauer and von Wirén, 2020). We then examined seed yield and N-related agronomic traits and collected xylem sap at three developmental stages between flowering and pod filling. Therein, we analyzed N forms as proxy for their uptake and translocation to the shoot and assessed the impact of different N forms on CK translocation. Nitrogen partitioning among vegetative and generative shoot organs at the end of pod development was used as indicator for N retranslocation during plant senescence. Since these measures were taken in 15 cultivars with differential N efficiency, we also examined genotypic variation in these N form-related physiological and agronomic traits.

## Materials and Methods

### Plant Material and Growth Conditions

Seeds of the winter oilseed rape cultivars were kindly provided by different German breeding companies: 11091433, 12091707, and PBC029 (Syngenta Seeds, Bad Salzuflen, Germany); BCSNE001 and BCSNE002 (Bayer CropScience Raps, Grundhof, Germany); DSV-01 and DSV-02 (Deutsche Saatveredelung, Lippstadt, Germany); KWS_01 and KWS_02 (KWS SAAT, Einbeck, Germany); LG00-304E and LG02-228D (Limagrain, Peine, Germany); and NPZ012, NPZ208, PBC007, PBC015, and Alpaga (Norddeutsche Pflanzenzucht Hans-Georg Lembke, Holtsee, Germany). All respective genotype pairs provided by each breeder consisted of an N-efficient and an inefficient cultivar out of the companies’ contemporary breeding material. PBC029, PBC007, and PBC015 are part of the genetically diverse Pre-BreedYield collection, which was created by German oilseed rape breeders for the homonymous research project, grant number 0315964J, funded by the German Federal Ministry of Education and Research. Those three lines were identified in a previous field experiment as contrasting in N efficiency (PBC015, PBC029: N efficient; PBC007: N inefficient) based on their N concentration in the youngest leaf and their seed yield formation at medium N supply (Supplementary Figure 1).

Field experiments were conducted in two subsequent years (2012/13 and 2013/14) at the IPK Gatersleben, Saxony-Anhalt, Germany (latitude: 51.824, longitude: 11.270, 106 m above sea level, Ø ann. temperature: 9°C, Ø ann. precipitation: 486 mm). The predominant soil type at this location is loamy clay with a soil taxation rated to 85 points. Mineralized N (N_min_) in the soil was determined at the beginning of each spring until a depth of 60 cm by Agrolab, Oberdorla, Germany (2013: 18 kg N ha^–1^; 2014: 22 kg N ha^–1^). Weather conditions of the individual years were recorded by the local weather station (Supplementary Figure 2).

Seeds were sown in late August in 10 m × 3 m plots with a density of 30 seeds m^–2^. Winter survival was recorded in early spring of the respective following year by counting the number of plants in a 1 m^2^ frame in two positions of the plot. On population average, 18 plants m^–2^ established after winter in 2012/1013, while in 2013/2014 only 13.5 plants m^–2^ were present. Stand densities of individual genotypes are listed in Supplementary Table 1. Crop protection was undertaken according to local practice whenever needed.

### Nitrogen Fertilizer Treatments

According to the highest N demands during oilseed rape development, plants were fertilized in autumn with 20 kg N ha^–1^ and in spring with 40 kg N ha^–1^ in the rosette stage as well as with 60 kg N ha^–1^ during shoot elongation (Supplementary Figure 3). 120 kg N ha^–1^ represents a comparatively moderate N dose for oilseed rape (Wiesler et al., 2001b) but was given with the aim to spread physiological and yield responses among the genotypes of the population at slightly suboptimal N availability and appeared reasonably against the need for reducing N balance surpluses in oilseed rape production. Here, in autumn fertilizer was applied uniformly as ammonium sulfate in order to establish comparable starting conditions for all plants before winter. In spring, both fertilizations were given either as ammonium nitrate (“Ammonium nitrate” treatment; ammonium nitrate obtained from Yara, Sluiskil, Netherlands) or as urea + urease inhibitor (“Urea” treatment) to study the physiological and yield response of oilseed rape to different N forms. 1.13 g *N*-(*n*-butyl)-thiophosphoric triamide (NBPT; AGROTAIN, liquid formulation by Koch Agronomic Services, Wichita, KS, United States) per kg urea fertilizer (SKW Stickstoffwerke Piesteritz, Lutherstadt Wittenberg, Germany) was used to stabilize applied urea. One oilseed rape genotype, PBC015, was additionally grown without any N fertilization to serve as reference for the effect of N fertilization *per se*.

In 2014, nitrate, ammonium, and urea concentrations in the soil were determined before and after fertilizer application (Supplementary Table 2) according to the methods described in DIN 19746 (Blume et al., 2000). Extraction of soil samples was carried out with 1 N KCl (Kuderna et al., 1993). To stabilize urea during extraction, NBPT was added (40 mg L^–1^ extraction solution). After microfiltration, samples were analyzed using a Continuous Flow Analyzer San++ (Skalar Analytical B.V., Breda, Holland). Ammonium was determined based on the modified Berthelot reaction. Ammonia reacts with salicylate and dichloroisocyanurate under the catalytic action of nitroprusside forming an indophenol color complex whose absorption was measured at 660 nm. To determine nitrate, it was first reduced to nitrite using a column containing copper–cadmium. Nitrite was determined by diazotizing with sulfanilamide and coupling with *N*-(1-naphthyl)ethylenediamine dihydrochloride to form a colored azo dye which was measured at 540 nm. For the detection of urea, diacetylmonoxime/thiosemicarbazide reaction was used. Diacetylmonoxime reacts with urea to form a chromogen which was detected at 520 nm (Douglas and Bremner, 1970).

### Field Plot Design

Field trials were installed in two sets, which allowed harvesting samples during the vegetation period from one set while keeping the other intact for final seed harvest. Plots in every trial were arranged in an extended Latin rectangle (*n* = 4) to correct data for soil differences in rows, columns, and quartiles. Every row, column, and quartile contained one ammonium nitrate- and one urea-fertilized plot per genotype.

### Determination of Seed Yield and Nitrogen Efficiency Parameters

As soon as mature seeds reached a moisture content of 12%, seeds were harvested with a combine from the core of each plot (7 m × 1.5 m). Seeds were dried to 7% moisture on a drying system (Hoopman Equipment & Engineering, Aalten, Netherlands). After cleaning with a grain sample cleaner (Baumann Saatzuchtbedarf, Waldenburg, Germany), seed yield per plot was weighed. Seed yield per area was determined by taking the plant number per square meter (Supplementary Table 1) into account.

NupE was calculated by dividing the sum of N accumulated in all aboveground shoot fractions by the sum of given N and soil N_min_ pools, both at an areal scale.

NutE was calculated by dividing the seed yield by the sum of N accumulated in all aboveground organ fractions, both at an areal scale.

NUE was calculated by dividing the seed yield by the sum of given N and soil N_min_ pools, both at an areal scale.

### Harvest of Xylem Sap From Field-Grown Rapeseed Plants

At three developmental stages, namely, at the end of flower development—BBCH57, mid of flowering—BBCH65, and mid of pod development—BBCH75 (according to Lancashire et al., 1991), xylem sap was collected from plants in either experimental year. However, due to differences in the temperature profile (Supplementary Figure 2), plant development differed between the two years. Thus, there were different time spans between xylem sap harvest and the preceding fertilization event. These time spans are indicated in Supplementary Figure 2.

Ten plants per plot were cut 2 cm above the soil surface with a scalpel early in the morning. A silicon tube, fitting to the diameter of the stem, was placed on top and closed with aluminum foil to avoid degradation of xylem sap components by UV light and to protect the xylem sap from dust and evaporation. On dry sampling days, the soil around each examined plant was watered with 1 l tap water to improve water availability to roots for adequate generation of root pressure. The first 100 μl of exudate was removed from the sampling tube due to contamination by cytosol as verified in pretests with cytosolic malate dehydrogenase activity. After 1–1.5 h, xylem sap of ten plants per plot was pooled. The exact sampling time was noted to determine the exudation rate. The exudate was frozen at −80°C.

### Extraction and Determination of Cytokinins, Amino Acids, Nitrogen Forms, and Total Nitrogen in Xylem Exudates

CKs were extracted and determined as described by Eggert and von Wirén (2017). Using OASIS^®^ HLB columns (Waters Corporation, Milford, MA, United States), CKs were cleaned up and extracted from xylem sap. Columns were attached to a vacuum chamber and conditioned with 1 ml acetonitrile and 1 ml methanol. Then, it was equilibrated by 1 ml 1% acetic acid. 500 μl xylem sap sample was acidified with 500 μl 2% acetic acid, spiked with internal CK standards for IP, cZ, Z, IPR, *cis*-zeatin riboside (cZR) and *trans-zeatin* riboside (ZR; marked with deuterium oxide or heavy N; OlChemIm, Olomouc, Czech Republic), gently mixed, and centrifuged for 10 min (4°C, 20800 × *g*) before loading it onto the column (according to extensive pretests of xylem sap from field-grown oilseed rape, we excluded the occurrence of other CK forms in the samples, i.e., dihydrozeatin, dihydrozeatinriboside, benzyladenine, benzyladenosine, Z-*O*-glucoside, Z-9-glucoside, Z-*O*-glucoside riboside, and IP-9-glucoside). Xylem sap samples were washed with 1 ml 2% ammonia and 1 ml 1% acidic acid. Finally, 1.7 ml 80% acetonitrile with 1% acidic acid (v/v) was used to elute CKs from the columns. Eluted samples were evaporated using a vacuum centrifuge (Martin Christ Gefriertrocknungsanlagen, Osterode, Germany) and reconstituted in 50 μl mobile phase. Therefore, 10 μl 50% methanol containing 0.5% formic acid (v/v) was added to the samples, gently mixed, and sonicated (ultrasonic bath: Bandelin electronic, Berlin, Germany) for 3 min. After adding 40 μl deionized water, samples were again mixed and sonicated as described before and centrifuged for 10 min (4°C, 20800 × *g*). CKs in the supernatant were separated by ultra-performance liquid chromatography (UPLC; Acquity System, Waters, Eschborn, Germany) using a BEH C18 column (Acquity UPLC BEH C18, 1.7 μm, 2.1 × 50 mm, Waters, Eschborn, Germany), which was heated to 40°C. As mobile phase A, LC-MS-grade water with 0.1% formic acid (v/v) was used. Mobile phase B was acetonitrile with 0.1% formic acid (v/v) to elute CKs from the column. The mobile phase gradient was as follows: 0 min—90% Eluent A + 10% Eluent B, 2 min—85% Eluent A + 15% Eluent B, 4.7 min—40% Eluent A + 60% Eluent B, 5 min—1% Eluent A + 99% Eluent B, 6 min—90% Eluent A + 10%. One run lasted for 7 min at 0.4 ml min^–1^ flow rate. Samples were ionized with electrospray ionization (ESI) positive ion mode and detected using a triple-quadrupole mass spectrometer (MS; Xevo TQ, Waters, Eschborn, Germany). Using the MassLynx Mass Spectrometry software (Waters, Milford, MA, United States), CK amounts were quantified based on an external CK standard curve ranging from 2.5 to 1000 nM for IP, cZ, Z, IPR, and cZR.

Analysis of AAs and ammonium in xylem exudates was performed as described by Hilo et al. (2017). AAs and ammonium were derivatized with aminoquinolyl-*N*-hydroxysuccinimidyl carbamate (ACQ)—a fluorescent agent which reacts with the amino group and forms stable urea-like structures (after Cohen and Michaud, 1993). 3 mg ACQ (prepared at IPK Gatersleben) was dissolved in 1 ml acetonitrile. 20 μl ACQ solution was used to derivatize AAs and ammonium in a 20-μl xylem sap sample in 160 μl 0.2 M boric acid, pH 8.8, for 10 min at 55°C. Separation of the ACQ-derivatized AAs and ammonium was performed using UPLC (Acquity H-Class system, Waters, Eschborn, Germany) equipped with a C18 reversed-phase column (ACQ Tag Ultra C18, 1.7 μm, 2.1 × 100 mm, Waters, Eschborn, Germany). Mobile phases were produced from “Eluent A Concentrate” and “Eluent B” for AAs analysis from Waters (Eschborn, Germany). One part of eluent A (pure “Eluent A Concentrate” from Waters, Eschborn, Germany) and nine parts of eluent C (pure “Eluent B” from Waters, Eschborn, Germany) were used to equilibrate the column. The mobile phase gradient consisted of four eluents: A, B (one part “Eluent B” from Waters, Eschborn, Germany, and nine parts LC-MS water from Th. Geyer, Renningen, Germany), C and D (LC-MS grade water from Th. Geyer, Renningen, Germany). The gradient during the run was as follows: 0 min—10% A + 90% C, 0.29 min—9.9% A + 90.1% C, 5.49 min—9% A + 80% B + 11% C, 7.10 min—8% A + 15.6% B + 57.9% C + 18.5% D, 7.69 min—7.8% A + 70.9% C + 21.3% D, 7.99 min—4% A + 36.3% C + 59.7% D, 8.68 min—10% A + 90% C. A run lasted 10.2 min at 0.7 ml min^–1^ flow rate. The column was heated to 50°C. AAs and ammonium were detected by a fluorescence detector (Acquity UPLC Photodiode Array eλ Detector, Waters, Eschborn, Germany) at 266 nm excitation and 473 nm emission wavelength and quantified by Empower Pro Software (Waters, United States) using a mixture of standards with different concentrations.

To determine nitrate concentrations, 5 μl of xylem sap was mixed with 160 μl 1% silicic acid in sulfuric acid (95–97%) and incubated on ice for 20 min. 1800 μl 3 M sodium hydroxide was added, and absorption at 410 nm was detected spectrophotometrically (Thermo Fisher Scientific, Waltham, MA, United States) to be quantified against a calibration curve (Wiseman and Jacobson, 1965).

Urea was determined according to Kojima et al. (2007), whereby the extraction step from plant tissue was omitted. Instead, 240 μl xylem sample was directly heated for 15 min at 99°C in the color development reagent containing 4.6 mM diacetylmonoxime, 1.28 mM thiosemicarbazide, 6.6% sulfuric acid, 14.6 μM ferric chloride hexahydrate, and 0.006% orthophosphoric acid. The samples were incubated. At 540 nm, the absorption was determined spectrophotometrically (Thermo Fischer Scientific, Waltham, MA, United States); the urea concentration was evaluated based on a standard curve.

For total N, 300 μl of xylem sap was lyophilized. N contents were determined using an elemental analyzer (EuroEA3000, Hekatech, Wegberg, Germany) and quantified based on 2.5-bis(5tert-butyl-2-benzo-oxazol-2-yl)thiophen (BBOT) standard with the Callidus software (Hekatech, Wegberg, Germany).

### Analysis of Aboveground Plant Nitrogen Allocation

At the end of pod development—BBCH79—two plants with representative shoot morphology for the whole plot were chosen and cut close to the soil surface and divided into vegetative (stem, branches, youngest leaves derived from branches, green leaves from stem, senescent leaves from stem) and generative (pods derived from stems, pods from branches) organs, pooled from both plants. The rather loose stands in our experiment allowed assigning detached leaves to individual plants, so that those could be collected from the ground.

Plant material was dried for 4 days at 80°C and weighed. The samples were ground (mill from Retsch, Haan, Germany), and N contents were determined using an elemental analyzer (EuroEA3000, Hekatech, Wegberg, Germany) and quantified based on BBOT standard with the Callidus software (Hekatech, Wegberg, Germany).

### Data Analysis

Before statistical analyses, data were corrected for soil heterogeneities at row, column, and quartile scales using the following formula:

$$V\,a\,l\,u\,e_{p\,l\,o\,t\,c\,o\,r\,r\,e\,c\,t\,e\,d}=v\,a\,l\,u\,e_{p\,l\,o\,t}-m\,e\,a\,n_{b\,l\,o\,c\,k}+m\,e\,a\,n_{w\,h\,o\,l\,e\,f\,i\,e\,l\,d\,t\,r\,i\,a\,l}.$$

For statistical analyses, the program SigmaPlot 11.0 (Systat Software, Erkrath, Germany) was used. Gaussian distribution was checked by Shapiro–Wilk test at *p* > 0.05. For normally distributed data, two groups were compared by unpaired *t*-test, multiple groups by ANOVA using Tukey’s test as *post hoc* test, and correlations were calculated by Pearson product moment correlation. For non-normally distributed data, two groups were compared by the Mann–Whitney rank-sum test, multiple groups on a monofactorial basis by Kruskal–Wallis one-way ANOVA plus Tukey’s test, and Spearman rank order was used for correlations. Non-Gaussian-distributed data for multifactorial analysis of variance were ranked manually. In figure or table legends, such cases are labeled by “ANOVA on ranks.”

## Results

### Yield and Nitrogen Use Efficiency in Response to Ammonium Nitrate- and Urea-Based Nitrogen Fertilization

**FIGURE 1:**
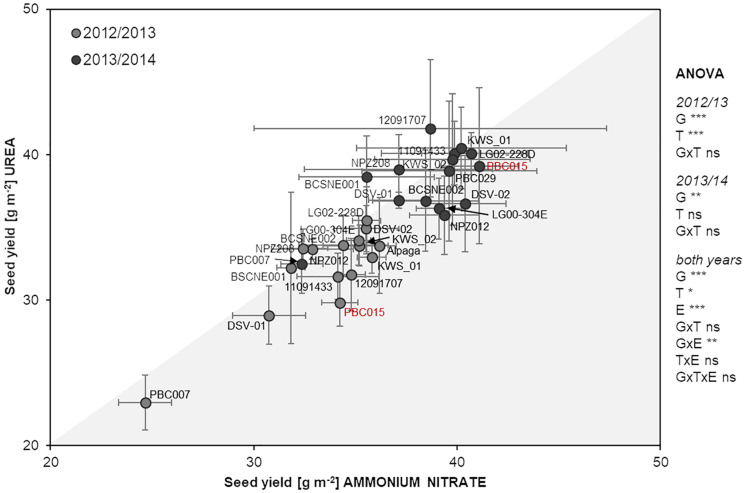
Seed yield of 15 oilseed rape genotypes after ammonium nitrate or urea fertilization in 2012/2013 and 2013/2014. Dots show means ± SD (*n* = 4). Asterisks *, **, and *** indicate significant differences or interactions at *p* < 0.05, *p* < 0.01, *p* < 0.001, resp., according to ANOVA on ranks; G, genotype; T, N treatment; E, environment; ns, not significant.

First, we examined the impact of N fertilization *per se* on seed yield formation in oilseed rape by growing one reference genotype, PBC015, without any N application. In a previous experiment, this genotype was characterized in a genetically diverse oilseed rape collection as N-efficient based on its high seed yield coupled with low N concentration in the youngest leaf (Supplementary Figure 1). Without fertilizer N input, PBC015 reached a seed yield of 21.6 ± 6.7 or 15.0 ± 1.5 g m^–2^ in 2012/13 or 2013/14, respectively (mean ± SD; *n* = 4). Compared to the unfertilized variants of this genotype, ammonium nitrate application led to a significant yield gain of ∼60% or even 170% in 2012/13 or 2013/14, respectively, while urea significantly increased the seed yield by ∼40% or 160% in the respective years according to Tukey’s test at *p* < 0.05. With about 30–34 or 39–41 g m^–2^, the seed yield of fertilized PBC015 in 2012/13 or 2013/14, respectively, was relatively close to the mean seed yields reported for oilseed rape in those years in Germany^1^, namely, 39.5 or 44.8 g m^–2^ (Figure 1). This indicates that at our experimental site moderate N fertilization of 120 kg N ha^–1^ was sufficient to reach the average yield level in Germany.

When we applied N as ammonium nitrate or urea, we found that the majority of the lines produced in trend, but not significantly, higher seed yield under ammonium nitrate supply. This resulted in overall significant higher seed yield of the whole population. About half of these lines showed a similar trend in the following year (e.g., LG00-304E), while other lines responded with higher yield formation under urea nutrition (e.g., 12091707; Figure 1). Over both years, ANOVA confirmed that differential yield formation among cultivars strongly depended on the environment. However, at lower significance (*p* < 0.05) the N treatment had an impact on yield formation of the population as well. This effect was mainly driven by the higher seed yield under ammonium nitrate nutrition in 2012/13 but could not be broken down to significant alterations in seed yield formation of individual genotypes.

We then asked the question if higher seed yield after ammonium nitrate fertilization translates also into higher NUE. Indeed, in 2012/13, when most genotypes tended to produce higher seed yield after ammonium nitrate application (Figure 1), NUE was significantly increased by a population mean of 1.2 kg seed yield per kg of applied N fertilizer (Table 1). Against our initial expectation, this difference appeared to mainly depend on NutE rather than on NupE, although this could not be confirmed at adequate significance. However, only 5 of the 11 genotypes with at least a trend to higher NUE under ammonium nitrate in 2012/13 showed a similar behavior in the second experimental year, when the overall response of the seed yield to the fertilized N form was weaker (Figure 1). Nonetheless, the population mean of NupE was significantly higher in ammonium nitrate-treated plants, i.e., by 10%, while NutE increased by 12% after urea application. Apparently, these differences compensated for each other when calculating final NUE. Notably, the higher NutE under urea nutrition was mainly based on harsh differences in two individual cultivars (BCSNE001 and BCSNE002), which did not respond consistently in the two years (Table 1). Taken together, ANOVA indicated a highly significant effect of the genotype on seed yield and NUE in either year, although this could not be broken down to the level of consistent responses of individual cultivars to the fertilized N form.

**TABLE 1:** Nitrogen-related agronomic parameters of 15 individual oilseed rape genotypes and as mean over all genotypes after ammonium nitrate or urea fertilization in the years 2012/13 and 2013/14.

Genotype	NupE [kg kg^–^^1^]			NutE [kg kg^–^^1^]			NUE [kg kg^–^^1^]		
	AN	Urea		AN	Urea		AN	Urea	
***2012/13***									
PBY007	0.9 ± 1.0	0.9 ± 0.3		19.9	21.3		20.9 ± 1.7	19.5 ± 0.6	C d
PBY015	0.8 ± 0.3	0.8 ± 0.1		34.2	29.3		29.0 ± 0.7	25.3 ± 2.8	* AB bc
Alpaga	1.0 ± 0.2	1.5 ± 0.5		29.6	19.9		30.7 ± 0.6	28.6 ± 4.4	A ac
11091433	1.2 ± 0.2	0.8 ± 0.3		24.3	30.8		28.9 ± 1.7	26.8 ± 1.3	AB ac
12091707	1.1 ± 0.1	0.9 ± 0.3		27.3	29.2		29.5 ± 0.6	26.9 ± 2.1	* AB ac
BCSNE001	1.0 ± 0.2	0.9 ± 0.3		26.3	29.7		27.0 ± 1.9	27.3 ± 1.8	AB ac
BCSNE002	1.0 ± 0.3	1.1 ± 0.3		30.7	25.9		29.2 ± 1.5	28.6 ± 1.7	AB ab
DSV-01	1.1 ± 0.5	1.3 ± 0.6		24.1	17.4		26.1 ± 1.3	24.5 ± 1.3	B c
DSV-02	1.2 ± 0.3	1.4 ± 0.4		25.2	21.3		30.1 ± 0.6	29.6 ± 0.9	AB a
KWS_01	1.1 ± 0.3	1.2 ± 0.4		27.2	24.4		30.3 ± 1.5	27.9 ± 1.2	A ac
KWS_02	1.3 ± 0.3	1.0 ± 0.4		23.1	29.7		29.8 ± 0.5	28.6 ± 1.4	AB ac
LG00-304E	0.8 ± 0.3	1.0 ± 0.3		38.9	28.4		29.8 ± 0.6	28.9 ± 2.0	AB ab
LG02-228D	1.3 ± 0.4	0.9 ± 0.1		23.9	31.9		30.1 ± 1.1	30.1 ± 1.6	A a
NPZ012	0.9 ± 0.3	0.9 ± 0.3		29.8	29.7		27.9 ± 1.0	28.4 ± 1.1	AB ac
NPZ208	1.1 ± 0.3	1.0 ± 0.2		25.4	28.3		27.5 ± 0.7	28.4 ± 1.4	AB ac
All lines	1.1 ± 0.3	1.1 ± 0.4		27.3	26.5		28.4 ± 2.7	27.2 ± 3.1	*
ANOVA	G ns, T ns, GxT ns			T ns			G ***, T ***, GxT ns		
***2013/14***									
PBY007	1.1 ± 0.1	1.0 ± 0.1	AB ab	24.4	26.9		26.5 ± 1.4	26.6 ± 2.2	B b
PBY015	1.1 ± 0.1	1.0 ± 0.1	AB ab	31.8	33.0		33.7 ± 3.5	32.2 ± 3.9	A ab
PBC029	0.9 ± 0.1	0.9 ± 0.1	B ab	35.0	32.9		33.1 ± 4.2	30.0 ± 2.3	AB ab
11091433	1.2 ± 0.2	1.1 ± 0.3	AB ab	26.2	28.8		32.7 ± 2.9	32.9 ± 1.8	AB ab
12091707	1.1 ± 0.1	1.0 ± 0.2	AB ab	29.8	33.9		31.7 ± 7.1	34.3 ± 3.9	AB a
BCSNE001	1.1 ± 0.2	0.8 ± 0.1	* AB b	30.4	37.8		32.5 ± 2.7	31.9 ± 2.3	AB ab
BCSNE002	1.0 ± 0.1	0.8 ± 0.1	AB b	28.8	38.2		29.1 ± 2.1	31.5 ± 2.9	AB ab
DSV-01	1.3 ± 0.2	1.1 ± 0.3	AB ab	24.2	27.1		31.5 ± 1.2	30.2 ± 0.5	AB ab
DSV-02	1.0 ± 0.2	0.7 ± 0.5	AB ab	31.1	34.4		30.4 ± 1.6	30.2 ± 2.7	AB ab
KWS_01	1.2 ± 0.1	1.2 ± 0.1	AB a	27.0	28.0		33.0 ± 3.1	33.2 ± 3.7	AB ab
KWS_02	1.3 ± 0.1	1.1 ± 0.1	AB ab	25.6	28.4		32.6 ± 0.9	32.5 ± 1.7	AB ab
LG00-304E	1.1 ± 0.1	0.9 ± 0.2	* AB ab	28.2	32.6		32.1 ± 2.0	29.7 ± 1.2	AB ab
LG02-228D	1.2 ± 0.1	1.1 ± 0.2	AB ab	27.2	30.2		33.4 ± 0.9	32.8 ± 1.7	AB ab
NPZ012	1.3 ± 0.1	1.0 ± 0.2	* A ab	24.8	30.8		32.3 ± 3.8	29.4 ± 1.9	AB ab
NPZ208	1.1 ± 0.1	1.2 ± 0.0	AB a	26.6	27.4		30.4 ± 1.6	32.0 ± 4.4	AB ab
All lines	1.1 ± 0.2	1.0 ± 0.2	*	28.1	31.4	*	31.7 ± 3.2	31.3 ± 3.0	
ANOVA	G ***, T ***, GxT ns			T *			G ***, T ns, GxT ns		

Table shows means ± SD (*n* = 4 biological replicates per genotype, *n* = 60 [over all genotypes]). No SD is shown for NutE since due to technical reasons aboveground N accumulation and seed yield of the same line had to be determined in parallel field plots. Asterisks show significant differences in N uptake efficiency (NupE), N utilization efficiency (NutE), or N use efficiency (NUE) under ammonium nitrate vs. urea treatment according to Mann–Whitney rank sum test at *p* < 0.05. Different upper- or lower-case letters indicate significant differences in NupE, NutE, or NUE among lines for the ammonium nitrate or urea treatment, resp., within one experimental year according to Tukey’s test on ranks at *p* < 0.05. *, **, and *** indicate significant differences or interactions at *p* < 0.05, *p* < 0.01, *p* < 0.001, resp., according to ANOVA on ranks. G, genotype; T, N treatment; ns, not significant; AN, ammonium nitrate.

### Impact of the Fertilized Nitrogen Form on Root-to-Shoot Translocation of Nitrogen and Cytokinins

**FIGURE 2:**
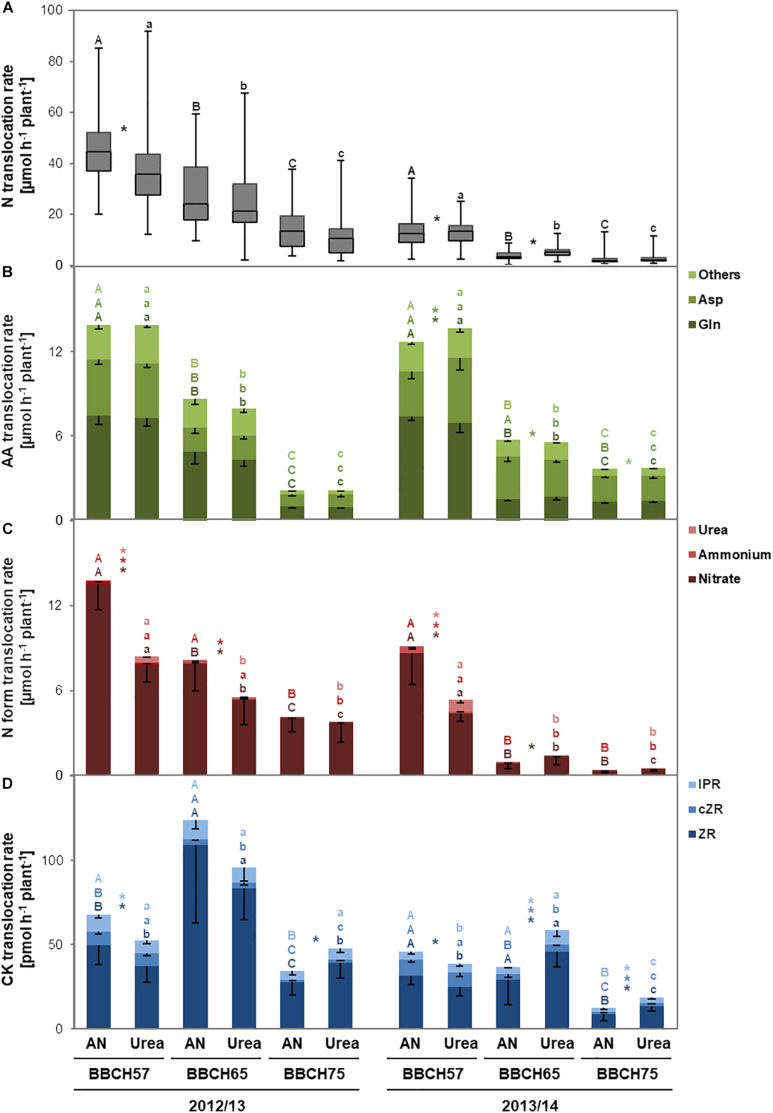
Mean translocation rates of total N **(A)**, amino acids (AAs, **B**), N forms **(C)**, and cytokinins (CKs, **D**) in the xylem sap of an oilseed rape panel at different developmental stages in dependence of ammonium nitrate or urea application. In the experimental years 2012/13 and 2013/14, 15 oilseed rape cultivars were grown for xylem sap analysis after fertilization with ammonium nitrate or stabilized urea (see Supplementary Figure 3). Boxes show median and first and third quartiles; whiskers show minimum and maximum of all data in a group (*n* = 60; **A**). Bars show means -SD (*n* = 60; **B,C**). Different upper- or lower-case letters show significant differences among the ammonium nitrate- or urea-treated variants, resp., within individual experimental years according to Tukey’s test on ranks at *p* < 0.05. Asterisks indicate significant differences between ammonium nitrate and urea treatment at individual developmental stages and years according to unpaired *t*-test (2012/13: Ammonium nitrate at BBCH57) or Mann–Whitney rank sum test (all other xylem compounds in both years and respective developmental stages) at *p* < 0.05. Letters and asterisks refer to the compound color code. AN, ammonium nitrate.

In order to answer the question if the increased seed yield and NUE observed in 2012/13 or at least the higher NupE in 2013/14 under ammonium nitrate are based on higher N uptake, we analyzed at three developmental stages translocation of total N and individual N forms in the xylem sap as proxy for N uptake (Peuke, 2010). In both years, we found that at BBCH57, shortly after applying 60 kg N ha^–1^ (i.e., after 2 days in 2012/2013 or after 11 days in 2013/14; Supplementary Figure 3), the population median of total N translocation in the xylem was higher in the ammonium nitrate treatment (Figure 2A). This was supported by 13 or 9 individual genotypes in 2012/13 or 2013/14, respectively, showing at least in trend or significantly a stronger response to ammonium nitrate over urea (Supplementary Table 3). At BBCH65 in 2012/13, i.e., 21 days after fertilizer application (Supplementary Figure 3), total N translocation halved, while the superior effect of ammonium nitrate was only observed in tendency (Figure 2A). In 2013/14, when 40 days had passed between fertilizer application and xylem sap collection, we observed the opposite effect as total N translocation was higher after urea application. At BBCH75, i.e., 40–48 days after fertilization, total N translocation further dropped to about 20% of the level at BBCH57 and was not affected by the N form anymore.

Total N was up to five times higher in 2012/13 than in 2013/14 (Figure 2A), pointing to a temporary higher N demand as plants in 2012/13 developed faster after a long cold period in early spring (Supplementary Figure 2). Nevertheless, in either year mean population AAs were translocated at similar rates of ∼14 μmol h^–1^ plant^–1^ at BBCH57 decreasing gradually to 2–3 μmol h^–1^ plant^–1^ at BBCH75 (Figure 2B). In general, higher total N translocation was mostly associated with a larger share of Gln while Asp became more important at lower N translocation, probably reflecting more efficient N translocation via the amide at ample N supply.

In both years, the population average of nitrate translocation at BBCH57 was 1.5- to 2-fold higher when ammonium nitrate was fertilized compared to urea; at later stages, this effect was only found at BBCH65 in 2012/13 (Figure 2C). Considering the significant share of nitrate in total N translocation, it appeared that total N translocation in the xylem was primarily affected by the N fertilizer form. Indeed, in 2014 analysis of N forms in the soil samples 7 days after fertilizer application confirmed that ammonium nitrate application led to 4- to 13-fold higher soil nitrate concentrations than the application of urea (Supplementary Table 2 and Supplementary Figure 3). Ammonium translocation showed the same dependency on growth stage and N fertilizer treatment as nitrate but at 20–50 times lower levels (Figure 2C). Urea translocation was only detectable at BBCH57, notably in all genotypes (Supplementary Table 3), suggesting that the absorption of fertilizer urea leads to significant levels of urea translocation in the xylem just shortly after urea application.

In the next step, we analyzed CK levels in the xylem sap to identify whether the fertilized N form may affect the phytohormonal balance in oilseed rape. We detected *trans*-zeatin-riboside (ZR) as a major translocated form together with *cis*-zeatin-riboside (cZR) and isopentenyl adenosine (IPR), the latter two forms conferring together between 5 and 30% of the overall cytokinin translocation rate (Figure 2D). Thereby, every CK form showed distinct developmental stage-depended translocation patterns: While ZR mostly increased between BBCH57 and BBCH65 before decreasing to or below initial values, cZR gradually decreased from BBCH57 to BBCH75. Translocation rates of IPR decreased at BBCH75. Especially at BBCH57, we found consistently in both years a larger population average of ZR when ammonium nitrate was applied compared to urea. Interestingly, at later plant development, i.e., at BBCH75 in both years and at BBCH65 in 2013/14, urea fertilization stimulated ZR as well as cZR and IPR translocation in the xylem sap of oilseed rape. This suggests a strong impact of ammonium nitrate on xylem CK translocation whenever the fertilizer is applied shortly before xylem sap sampling, whereas urea promotes CK translocation when the period between fertilization and sampling became longer.

While the N forms in the xylem were mostly not affected by the genotype, significant genotypic differences existed in CK forms especially when plants were supplied with ammonium nitrate fertilizer (Supplementary Table 3). Shortly after ammonium nitrate application, i.e., at BBCH57 in either year, highest (LG00-304E—ZR, DSV-02—cZR) and lowest (11091433—IPR) CK translocation rates were observed repeatedly in the same cultivars, pointing to a considerable impact of the genotype on its responsiveness to the fertilized N form.

### Impact of the Fertilized Nitrogen Form on Aboveground Nitrogen Partitioning

**FIGURE 3:**
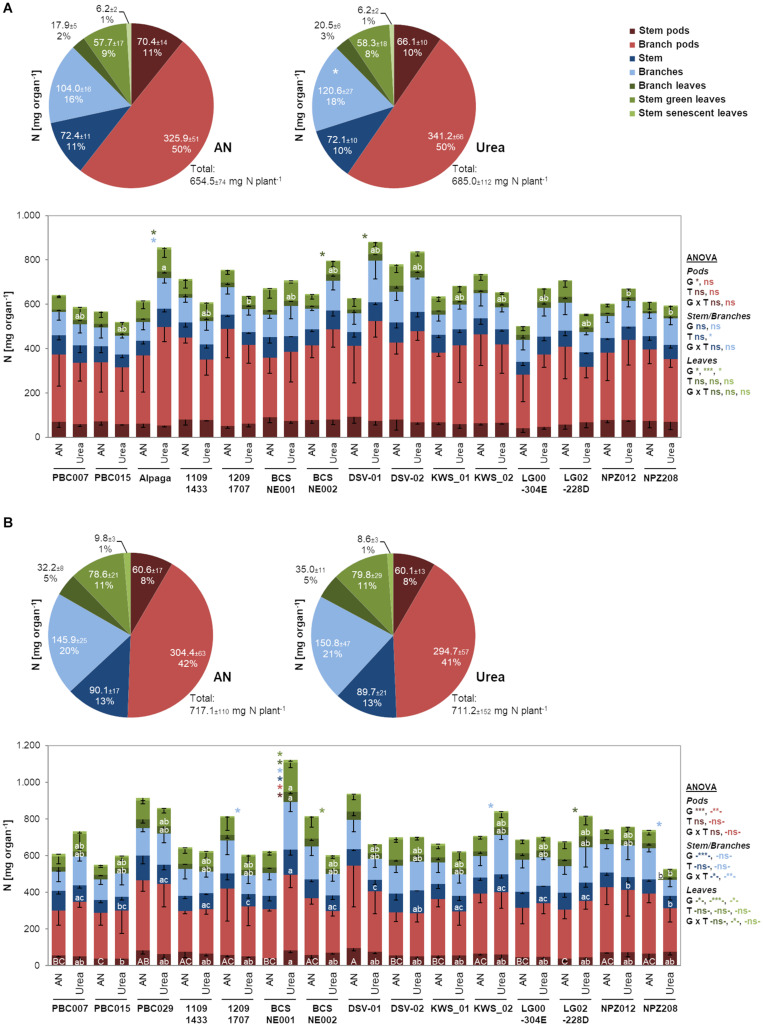
Individual N pools in aboveground organs and organ fractions as mean over all genotypes or in individual genotypes after ammonium nitrate or urea fertilization in 2012/13 **(A)** and 2013/14 **(B)**. Pie charts show N contents (means of all lines ± SD) of the indicated organs and the corresponding percentage to total plant N at BBCH79 (*n* = 60). Asterisks in the right-hand chart indicate significant mean or median differences among ammonium nitrate and urea treatment according to unpaired *t*-test (**A:** stem pods, branch pods) or Mann–Whitney rank sum test (**A:** all other fractions, **B:** all fractions) at *p* < 0.05. Bars show means of single lines -SD (*n* = 4). ANOVA or ANOVA on ranks (- -) results *, **, and *** indicate significant differences or interactions at *p* < 0.05, *p* < 0.01, and *p* < 0.001, respectively; G, genotype; T, N treatment; ns, non-significant. Asterisks referring to the organ color code indicate significant mean difference among ammonium nitrate and urea treatment within a line according to unpaired *t*-test at *p* < 0.05. Different upper/lower case letters within a bar indicate significant mean or median differences among the lines within ammonium nitrate/urea treatment according to Tukey’s test (**A**—*Ammonium nitrate:* stem pods, stem, branches, branch leaves, stem senescent leaves; - *Urea:* stem pods, branch pods, stem, branches; **B**—*Ammonium nitrate:* stem pods, stem green leaves; - *Urea:* stem pods, branch pods, stem) or Tukey’s test on ranks (**A**—*Ammonium nitrate:* branch pods, stem green leaves; - *Urea:* branch leaves, stem green leaves, stem senescent leaves; **B**—*Ammonium nitrate:* branch pods, stem, branches, branch leaves, stem senescent leaves, - *Urea:* branches, branch leaves, stem green leaves, stem senescent leaves) at *p* < 0.05. AN, ammonium nitrate.

Since both the form of translocated N and the associated changes in CKs may alter N assimilation pathways and even N partitioning among aboveground organs, we investigated N allocation to stems, leaves, and pods and even further differentiated these organs according to their position and age. Regarding the total N determined in these seven fractions or pools, there were no significant differences between ammonium nitrate- and urea-fertilized plants in 2012/13, indicating that total N uptake per plant was not affected by the fertilized N form (Figure 3A). When comparing the sizes of the N pools in the different organs, we found highly coherent values in the two N treatments. However, larger N pools in branches and in trend also in their pods after urea supply suggested that urea may favor total N delivery and partitioning to branch-associated organs. We then looked for genotypic differences in N partitioning under ammonium nitrate- or urea-based nutrition. Indeed, in 3 out of 15 cultivars, there was higher N allocation to the leaf fraction attached to branches. We then verified this observation in the subsequent year 2013/14, when total N was only slightly higher. Here, N partitioning to the major N pool of branch pods was 8–9% lower, whereas more N was retained in leaves, irrespective of their position (except for senescent ones; Figure 3B). Also in the second year, when more N was retained in vegetative organs, N partitioning among organs remained almost the same under ammonium nitrate and urea nutrition. Thus, there were no consistent differences in the responsiveness of N pool sizes to the fertilized N form in the two years. However, considering ANOVA, there was an overall effect of the genotype on N partitioning, which was influenced by the year but largely independent of the fertilized N form. Against our expectation, these results indicated that ammonium nitrate-fertilized oilseed rape does not retranslocate N later from vegetative to the generative organs suggesting that senescence was not retarded.

## Discussion

### Response of Seed Yield and Nitrogen Use Efficiency to the Fertilizer Nitrogen Form Is Subject to Genotypic and Environmental Variation

Against the relatively consistent short-term impact of ammonium nitrate on xylem CK and N translocation, yield effects by differential N supply were strongly dependent on the environmental conditions of a year (Figure 1 and Table 1). Nevertheless, ammonium nitrate had in trend an overall positive effect on seed yield formation compared to urea, but this was mainly driven by the growth response in 2012/13. In that year, a long cold period lasted until March 2013 (Supplementary Figure 2), leading to accelerated plant development thereafter and shortening the time between N fertilization and seed development. Thus, ammonium nitrate-stimulated N delivery to the shoot could hold on until post-flowering development (BBCH75; Figure 2), thereby potentially improving pod formation and seed development (Diepenbrock, 2000). The importance of post-flowering N uptake for NUE in oilseed rape (Ulas et al., 2012) was reflected by overall higher NUE after ammonium nitrate supply in our experiment (Table 1). Broken down into components, the NupE in that year was not higher after ammonium nitrate compared to urea fertilization. There was rather a trend for better NutE under ammonium nitrate (Table 1). In general, NutE becomes increasingly important for NUE under sufficient or elevated N supply (Berry et al., 2010; Schulte auf’m Erley et al., 2011; Kessel et al., 2012).

In 2013/14, which showed a more typical temperature course for Germany (Supplementary Figure 2), NUE of the oilseed rape lines was not influenced by the N fertilizer form anymore (Table 1). As we expected initially, ammonium nitrate improved NupE in that year, while urea led to better NutE, but these effects compensated for each other when calculating total NUE. Moreover, only less than half of the genotypes showed the same trend for yield gain under ammonium nitrate compared to the first experimental year (Figure 1). Confirmation of genotypic differences in the responsiveness to ammonium nitrate in this handful of lines requires further studies.

### Xylem-Translocated Nitrogen and Cytokinin Forms Are Modulated by Ammonium Nitrate and Urea Nutrition

With the present study, we addressed the hypothesis whether relative to urea an ammonium nitrate-based N nutrition of oilseed rape (i) increases overall N uptake and (ii) stimulates the production of CKs in roots as well as their root-to-shoot translocation. By assessing xylem sap at different developmental stages, we found that ammonium nitrate as fertilized N form stimulated the translocation of both ammonium and nitrate, even after a period up to 21 days between fertilizer application and xylem sap sampling (BBCH65 in 2012/13; Figure 2 and Supplementary Figure 3). As nitrate is mainly assimilated in shoots whenever sufficient N is available (Andrews, 1986), the increased shoot-ward transport directly reflects root uptake and xylem loading. In contrast, ammonium assimilation takes place primarily in roots (Finnemann and Schjoerring, 1999), explaining the low abundance of ammonium in the xylem sap. In addition, it must be taken into consideration that most likely a considerable fraction of fertilized ammonium became immobilized by adsorption to the soil matrix, further decreasing ammonium uptake and recovery in the xylem sap. Large amounts of ammonium-N detected in the soil samples (Supplementary Table 2) most likely overestimated plant-available ammonium, because ammonium was extracted with the strong extraction agent KCl that also desorbs ammonium bound to the soil matrix (Kirschke et al., 2019). Urea fertilization stimulated the translocation of urea as N form whenever xylem sap was sampled shortly after fertilization (Figures 1, 2 and Supplementary Figure 3), which relies on low-affinity and especially high-affinity urea transport systems (Kojima et al., 2007), whose transport capacities are lower than those for ammonium or nitrate (Tan et al., 2000; Arkoun et al., 2012). In all genotypes, urea translocation could be observed up to 11 days after fertilization (Figure 1 and Supplementary Table 3). As confirmed by urea detection in the soil solution only after urea supply (Supplementary Table 2), this indicates that urea was available and taken up at considerable rates only during the short period of excess urea over ammonium and nitrate in the soil. Indeed, the co-applied urease inhibitor NBPT, a structural analog of urea, is known to delay urea hydrolysis for about 10–14 days in soils (Watson, 2005). Immediately after urea application, total N translocation in the xylem decreased (Figure 2), probably as a result of comparatively lower soil nitrate levels (Supplementary Table 2) and the repressive effect of urea on the transcriptional regulation the nitrate transporters *BnNRT1.1* and *BnNRT1.2* (Arkoun et al., 2012). In 2013/14, urea uptake and translocation were associated with higher abundance of Asp relative to Gln (Figure 2), although the latter is the more N-efficient translocation form of organic N (Schlatter and Langer, 2008), indicating that the urea fertilizer was temporarily less efficient than ammonium nitrate in meeting the N demand of oilseed rape. This observation supports the proposition by Barneix and Causin (1996) that the AA composition in the xylem may act as messenger for root-to-shoot transmission of the root N status.

Z-type CKs have been described to be mainly synthesized in roots, at least in *Arabidopsis thaliana*, as the required monooxygenase CYP735A is predominantly expressed in roots (Takei et al., 2004b). In contrast, IP-type CKs are mainly synthesized in shoots in response to nitrate (Sakakibara, 2006), while cZ-type CKs are generally less affected by the plant nutritional status (Schäfer et al., 2015). Accordingly, we found ZR being the most abundant CK form in the xylem followed by IPR (Figure 2). In both years, cZR had its highest share at BBCH57 suggesting that this CK form loses functional importance at later developmental stages. Higher translocation rates of CKs after ammonium nitrate fertilization, especially at early sampling time points (Figure 2D), are in agreement with xylem sap analyses from hydroponically grown barley plants, where major CK forms were determined by radio immune-assay (Bauer and von Wirén, 2020). There, CK translocation rates decreased gradually when N supplementation in form of nitrate was replaced by urea.

When we analyzed xylem sap 40–48 days after fertilizer application (corresponding to BBCH75 in 2012/13 and BBCH65 and BBCH75 in 2013/14), we observed that CK translocation in the xylem increased in the urea treatment (Figure 2). In 2013/14, nitrate translocation rates increased after urea fertilization as well, thus increasing total N export to the shoot 40 days after urea fertilization (BBCH65). Obviously, fertilized urea ensures a longer-lasting N availability for oilseed rape, whereas ammonium nitrate-derived N depleted earlier. Preventing early urea hydrolysis by urease inhibitor amendment and thus indirectly delaying nitrification of ammonium may explain at later stages the high abundance of nitrate after urea fertilization. Interestingly, at BBCH75, when total N uptake declined during the course of plant senescence (Malagoli et al., 2004), urea fertilization still stimulated CK translocation (Figure 2). Nitrate as well as ammonium can promote CK synthesis in roots (Kamada-Nobusada et al., 2013). Based on consistently positive correlations between nitrate and CK translocation rates (Supplementary Figure 4), we conclude that higher CK levels in the xylem primarily reflect a signaling response to nitrate and to a certain extent also to ammonium, which in case of urea fertilization occurs after urea hydrolysis. However, since seed yield tended to be higher under ammonium nitrate supply than under urea, we speculate that higher xylem CK levels before (BBCH57) and during flowering (BBCH65) may be more beneficial for yield formation in oilseed rape than after flowering (BBCH75). Applying different doses of synthetic CKs externally to the xylem sap during those developmental stages could improve understanding of the importance of temporary CK dynamics in the xylem for yield formation in future experiments.

### Influence of the Fertilizer Nitrogen Form on the Distribution of Nitrogen Between Vegetative and Generative Shoot Organs

Initially, we expected that ammonium nitrate fertilization may stimulate N uptake due to the higher uptake capacity for ammonium nitrate vs. urea (Tan et al., 2000; Arkoun et al., 2012). Although consistently larger amounts of N were transported to the shoot after ammonium nitrate fertilizer application at flower development and even at flowering in 2012/13 (Figure 2), we did not find significant differences in total aboveground N accumulation at BBCH79 in either year (Figure 3). Arkoun et al. (2012) found that among ammonium sulfate, ammonium nitrate, non-stabilized urea, and stabilized urea, the latter was most effective for shoot N accumulation. On the other hand, Fismes et al. (2000) found contrasting trends for shoot N accumulation when applying nitrate or urea to oilseed rape. Most likely, shoot N accumulation in response to different fertilizer N forms depends on environmental and experimental conditions. Our study provides evidence that ammonium nitrate led to better N provision shortly after fertilizer application, while in the urea treatment N delivery to the shoot set in later but held on for longer. Accordingly, urea-treated plants allocated more N to those organs that are typically built later during plant development, namely, to branches and branch-associated leaves and pods (Figure 3).

In the ammonium nitrate treatment, we further expected a delay in plant senescence due to the stronger stimulation of CK translocation by nitrate (Jibran et al., 2013; Bauer and von Wirén, 2020), which may express in a lower amount of N retranslocated from vegetative to generative organs. However, assessing the N pool sizes in aboveground plant organs did not confirm this expectation (Figure 3). While in the second year more N remained in vegetative shoot organs than in the first year, there was no detectable influence of the applied fertilizer N form on the allocation of N to generative (pod) relative to vegetative fractions.

In summary, we confirmed the hypotheses that ammonium nitrate-based nutrition promotes N uptake, at least shortly after fertilization, and increases CK synthesis and translocation, when compared to urea whose availability strongly depends on prevention of its hydrolysis in the soil. At later developmental stages when urea is converted, urea-derived N, mostly in the form of nitrate, is also able to increase N uptake and CK translocation to the shoot. Consequently, urea tends to increase N partitioning in the shoot toward generative organs, while total N accumulation in the shoot at the end of the vegetation period is not necessarily affected. Although ammonium nitrate-based fertilization exerted here an overall trend for yield gain in oilseed rape, this effect could not be broken down to consistent effects of the given N form on N-related efficiency parameters, since the impact of seasonal variations appeared too strong.

## Data Availability Statement

The original contributions presented in the study are included in the article/Supplementary Material, further inquiries can be directed to the corresponding author.

## Author Contributions

DH and NW conceived the study. DH and HH designed methodology. DH and HH collected the data. DH and NW analyzed the data. DH and NW wrote the manuscript with contribution of HH. All authors contributed to the article and approved the submitted version.

## Conflict of Interest

HH was employed by company SKW Stickstoffwerke Piesteritz GmbH. The remaining authors declare that the research was conducted in the absence of any commercial or financial relationships that could be construed as a potential conflict of interest.

## Footnotes

1 www.fao.org (accessed on 1.7.2020).

## Supplementary Material

Supplementary Figure 1 Nitrogen concentrations in the youngest leaf of the main shoot at BBCH69 **(A)** and seed yield **(B)** of genotypes from the Pre-BreedYield collection in 2011/12.

Supplementary Figure 2 Weather conditions during the experimental years 2012/13 **(A)** and 2013/14 **(B)** as recorded by the local weather station.

Supplementary Figure 3 Sampling and fertilization scheme in the field trials of the experimental years 2012/13 and 2013/14.

Supplementary Figure 4 Correlations between the translocation rates of total cytokinins (CK) with different N forms in the xylem sap of 15 oilseed rape genotypes at BBCH57, BBCH65, and BBCH75 in 2012/13 **(A)** and 2013/14 **(B)**.

Supplementary Table 1 Stand density of winter oilseed rape genotypes in 2012/13 and 2013/14.

Supplementary Table 2 Concentrations of ammonium, nitrate and urea in the soil before spring N fertilizer application or 7 days after applying N fertilizers at a dose of 40 kg N ha^–1^ or 60 kg N ha^–1^, respectively, in 2014.

Supplementary Table 3 Translocation rates of total N, amino acids, N forms and cytokinins in the xylem sap of 15 winter oilseed rape genotypes at the developmental stages BBCH57, BBCH65, and BBCH75 in the experimental years 2012/13 and 2013/14.
