# Supplementary material for: Seed Yield and Nitrogen Efficiency in Oilseed Rape After Ammonium Nitrate or Urea Fertilization
Source: Front Plant Sci. 2021 Jan 27;11:608785. doi: 10.3389/fpls.2020.608785 (PMC7874180; doi:10.3389/fpls.2020.608785)
Supplement: Supplementary Figure 1 — Nitrogen concentrations in the youngest leaf of the main shoot at BBCH69 (A) and seed yield (B) of genotypes from the Pre-BreedYield collection in 2011/12. [file Data_Sheet_1.PDF]

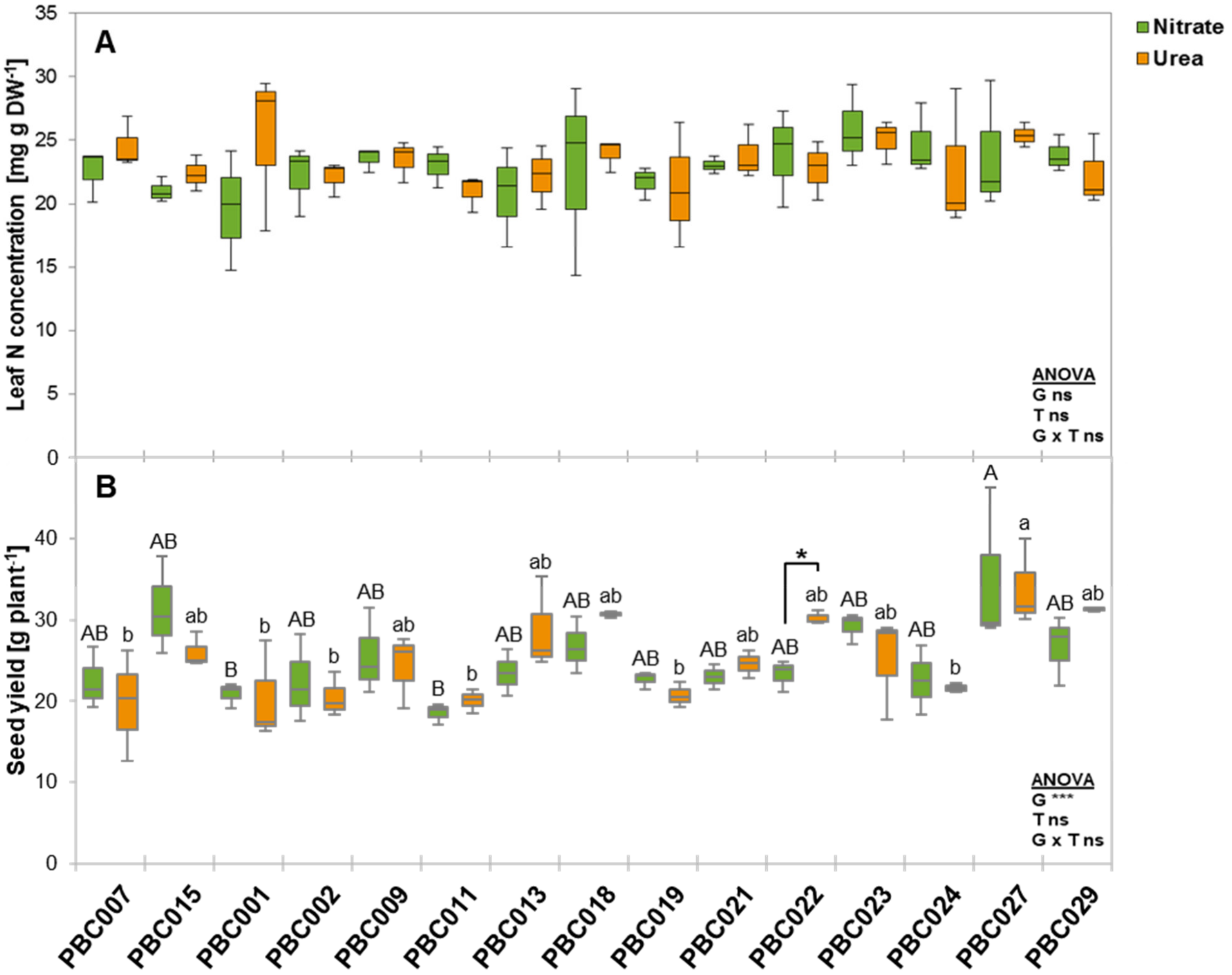

S1 Figure. N concentration in the youngest leaf of the main shoot at BBCH69 (A) and seed yield (B) of genotypes from the Pre-BreedYield* collection in 2011/12. Cultivation conditions and yield analyzes were as described in the Material and Methods section for the field trials in 2012/13 and 2013/14. Leaf material was taken at BBCH69 [1], dryed for 3 d at 60 °C and ground to determine N concentration using an elemental analyzer. Boxes show median, first and third quartile; whiskers represent minimum and maximum of all data in a group; n=3. Significant differences among ammonium nitrate- or urea-treated genotypes are shown by upper or lower-case letters, respectively, according to Tukey´s test at $p<0.05$. Asterisks indicate significant mean difference among ammonium nitrate and urea treatment within a genotype according to unpaired t-test at $p<0.05$. ANOVA results *, **, *** indicate significant differences or interactions at $p<0.05$, $p<0.01$, $p<0.001$, respectively; G=genotype, T=N treatment, ns=non-significant.

* The Pre-BreedYield collection was created by different German breeding companies as set of genetically diverse current and old adapted varieties as well as one resynthesized oilseed rape line from *B. rapa* and *B. oleracea*. It was used within the frame of the Pre-Breed Yield project (funded by the German Federal Ministry of Education and Research, grant number 0315964J).

1. Lancashire, P.D., et al., *A uniform decimal code for growth stages of crops and weeds.* Annals of Applied Biology, 1991. **119**(3): p. 561-601.
