# Supplementary material for: Seed Yield and Nitrogen Efficiency in Oilseed Rape After Ammonium Nitrate or Urea Fertilization
Source: Front Plant Sci. 2021 Jan 27;11:608785. doi: 10.3389/fpls.2020.608785 (PMC7874180; doi:10.3389/fpls.2020.608785)

S2 Figure. Weather conditions during the experimental years 2012/13 (A) and 2013/14 (B) as recorded by the local weather station. Longtime avarages include precipitation and temperature data from 1969-2014 recorded in Gatersleben. EXP = Precipitation/temperature during the respective experimental runtime.

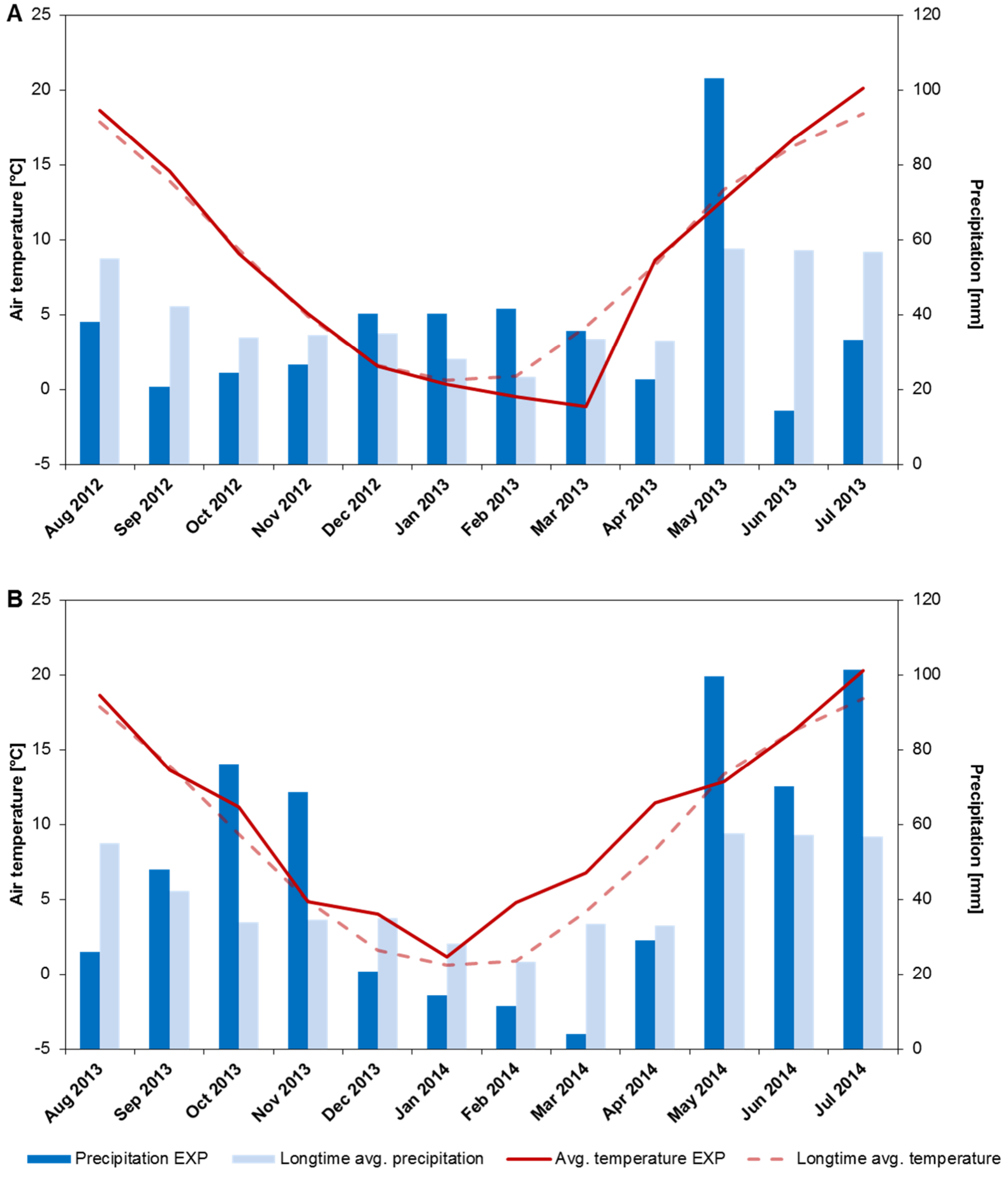

Supplement: Supplementary Figure 2 — Weather conditions during the experimental years 2012/13 (A) and 2013/14 (B) as recorded by the local weather station. [file Data_Sheet_3.PDF]
