# Supplementary material for: Seed Yield and Nitrogen Efficiency in Oilseed Rape After Ammonium Nitrate or Urea Fertilization
Source: Front Plant Sci. 2021 Jan 27;11:608785. doi: 10.3389/fpls.2020.608785 (PMC7874180; doi:10.3389/fpls.2020.608785)
Supplement: Supplementary Figure 3 — Sampling and fertilization scheme in the field trials of the experimental years 2012/13 and 2013/14. [file Data_Sheet_5.PDF]

**S3 Figure. Sampling and fertilization scheme in the field trials of the experimental years 2012/13 and 2013/14.** BBCH = phenological plant developmental stages according to the Federal Biological Agency, Federal Office for Plant Varieties and Chemical Industry [1]. * Fertilizer was applied as ammonium nitrate or urea treatment, $^{+}$ Denomination of soil sampling events accords to S2 Table.

| | | Period from last fertilization event to xylem sampling [d] | |
|---|---|---|---|
| BBCH | | 2012/13 | 2013/14 |
| 0 | Sowing | | |
| 16 | Fertilization: 20 kg N ha$^{-1}$ | | |
| 30 | Soil sampling „before N fertilization” $^{+}$<br>Fertilization: 40 kg N ha$^{-1}$ * | | |
| 52 | Soil sampling „7 d after 40 kg N ha$^{-1}$“ $^{+}$ | | |
| 55 | Fertilization: 60 kg N ha$^{-1}$ * | | |
| 56 | Soil sampling „7 d after 60 kg N ha$^{-1}$“ $^{+}$ | | |
| 57 | Harvest of xylem sap | 2 | 11 |
| 65 | Harvest of xylem sap | 21 | 40 |
| 75 | Harvest of xylem sap | 40 | 48 |
| 79 | Harvest of above-ground fractions | | |
| 99 | Seed harvest | | |


1. Lancashire PD, Bleiholder H, Boom TVD, Langelüddeke P, Stauss R, Weber E, et al. A uniform decimal code for growth stages of crops and weeds. Ann Appl Biol. 1991;119(3):561-601.
