# Supplementary material for: Seed Yield and Nitrogen Efficiency in Oilseed Rape After Ammonium Nitrate or Urea Fertilization
Source: Front Plant Sci. 2021 Jan 27;11:608785. doi: 10.3389/fpls.2020.608785 (PMC7874180; doi:10.3389/fpls.2020.608785)

S4 Fig. **Correlations between the translocation rates of total cytokinins (CK) with different N forms in the xylem sap of 15 oilseed rape genotypes at BBCH57, BBCH65 and BBCH75 in 2012/13 (A) and 2013/14 (B).** Components were correlated by Spearman rank order correlations. Values for r and p are shown separately for ammonium nitrate (green) and urea (orange) treatments (n=60); n.d. = not determined.

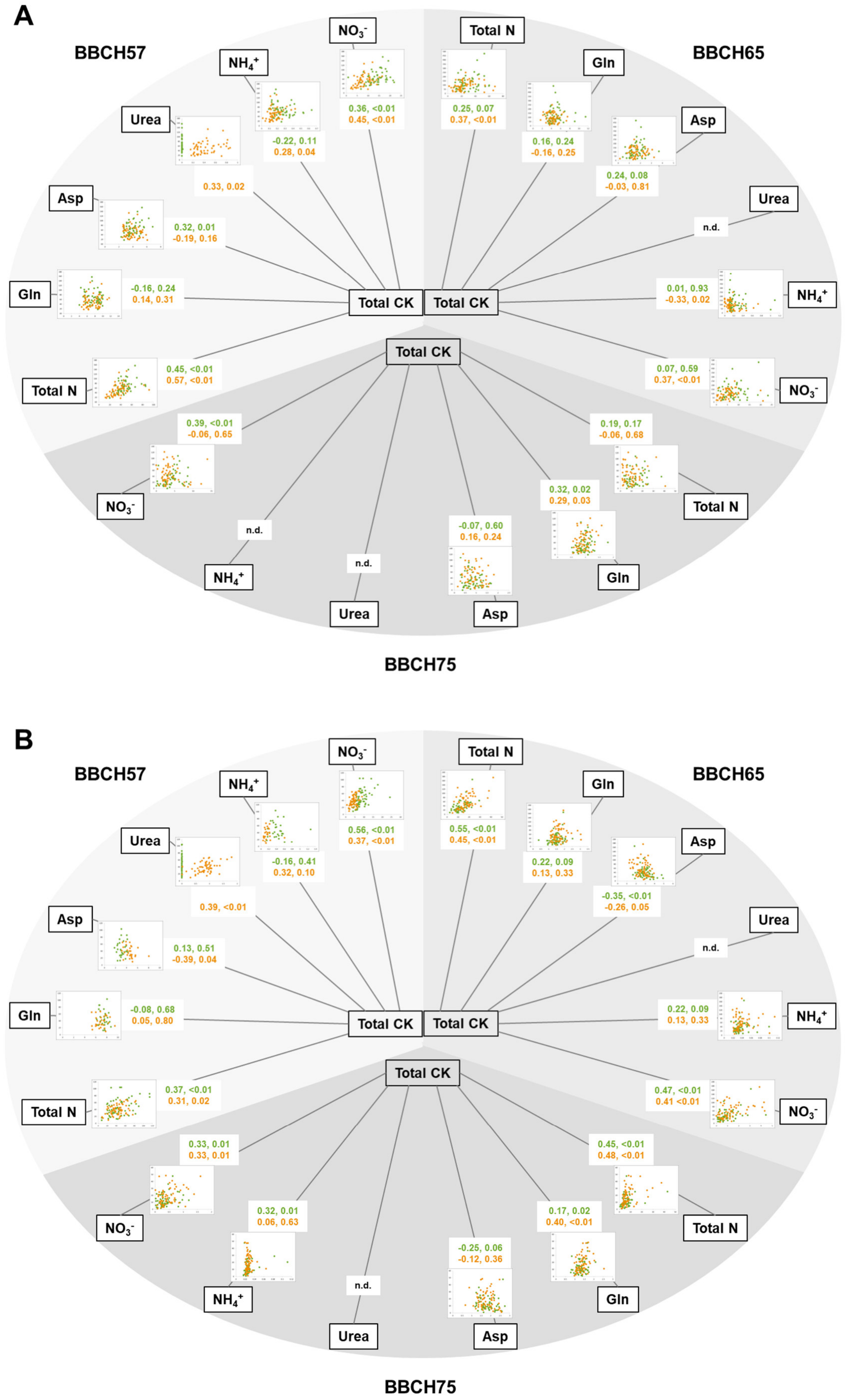

Supplement: Supplementary Figure 4 — Correlations between the translocation rates of total cytokinins (CK) with different N forms in the xylem sap of 15 oilseed rape genotypes at BBCH57, BBCH65, and BBCH75 in 2012/13 (A) and 2013/14 (B). [file Data_Sheet_7.PDF]
