# Supplementary material for: Seed Yield and Nitrogen Efficiency in Oilseed Rape After Ammonium Nitrate or Urea Fertilization
Source: Front Plant Sci. 2021 Jan 27;11:608785. doi: 10.3389/fpls.2020.608785 (PMC7874180; doi:10.3389/fpls.2020.608785)
Supplement: Supplementary Table 1 — Stand density of winter oilseed rape genotypes in 2012/13 and 2013/14. [file Data_Sheet_2.PDF]

S1 Table. Stand density of winter oilseed rape genotypes in 2012/13 and 2013/14. Table shows means ±SD (n=4 for single genotypes, n=60 for all genotypes). ANOVA results *, **, *** indicate significant differences or interactions at p<0.05, p<0.01, p<0.001, respectively; G=genotype, T=N treatment, ns=non-significant. Asterisks indicate significant mean difference among ammonium nitrate and urea treatment within a single genotype according to unpaired t-test at p<0.05. Different upper/lower case letters indicate significant mean differences among the genotypes within ammonium nitrate/urea treatment according to Tukey´s test at p<0.05. AN=Ammonium nitrate.

**Genotype**	**Stand density [plants m^{-2}]**					
	**2012/13**			**2013/14**		
	**AN**		**Urea**	**AN**		**Urea**
PBC007	20.0 ±3.0		18.8 ±0.9	14.2 ±1.6	AB	13.0 ±1.3
PBC015	19.5 ±2.4		18.6 ±1.8	12.1 ±3.3	AB	12.8 ±4.8
Alpaga	21.9 ±3.5		19.2 ±2.6	*not grown in 2013/14*		
PBC029	*not grown in 2012/13*			11.7 ±1.9	AB	12.8 ±1.8
11091433	19.1 ±3.3		18.8 ±3.3	13.6 ±2.7	AB	14.8 ±2.6
12091707	18.5 ±0.9		16.5 ±1.5	12.1 ±2.4	AB	13.5 ±2.1
BCSNE001	15.3 ±1.4		14.0 ±2.5	11.6 ±2.2	AB	11.2 ±1.4
BCSNE002	16.5 ±2.0		19.6 ±2.4	9.1 ±1.7	B *	11.8 ±1.0
DSV-01	19.7 ±2.4		16.9 ±3.9	14.0 ±0.3	AB	15.0 ±4.5
DSV-02	17.0 ±2.7		17.0 ±3.6	12.7 ±3.1	AB	11.2 ±0.7
KWS_01	16.1 ±3.0		17.6 ±1.3	15.8 ±2.7	A	15.5 ±1.3
KWS_02	17.8 ±4.3		19.0 ±2.1	14.6 ±1.6	AB	14.9 ±1.1
LG00-304E	16.6 ±1.6		18.6 ±2.8	14.8 ±0.6	A	12.4 ±1.4
LG02-228D	17.5 ±2.9		18.3 ±2.7	14.8 ±2.3	A	14.2 ±2.0
NPZ012	16.1 ±0.4	*	19.6 ±1.8	13.4 ±2.7	AB	12.4 ±2.4
NPZ208	18.0 ±3.3		20.7 ±2.9	15.0 ±1.7	A	14.2 ±1.1
All genotypes	18.0 ±2.9		18.2 ±2.7	13.3 ±2.6		13.3 ±2.4
**ANOVA**	G *, T ns, GxT ns			G ***, T ns, GxT ns		
