# Supplementary material for: Seed Yield and Nitrogen Efficiency in Oilseed Rape After Ammonium Nitrate or Urea Fertilization
Source: Front Plant Sci. 2021 Jan 27;11:608785. doi: 10.3389/fpls.2020.608785 (PMC7874180; doi:10.3389/fpls.2020.608785)
Supplement: Supplementary Table 2 — Concentrations of ammonium, nitrate and urea in the soil before spring N fertilizer application or 7 days after applying N fertilizers at a dose of 40 kg N ha–1 or 60 kg N ha–1, respectively, in 2014. [file Data_Sheet_4.PDF]

S2 Table. Concentrations of ammonium, nitrate and urea in the soil before spring N fertilizer application or 7 days after applying N fertilizers at a dose of 40 kg N ha^{-1} and 60 kg N ha^{-1}, respectively, in 2014. Soil samples labeled with "before N fertilization", "7 d after 40 kg N ha^{-1}" and "7 d after 60 kg N ha^{-1}" were taken at the oilseed rape developmental stages BBCH30, BBCH52 and BBCH56, respectively. "Control" = unfertilized soil.

Soil depth [cm]	N treatment	N forms [mg 100 g soil DW^{-1}]		
		NH_4^+	NO_3^-	Urea
Before N fertilization				
0-30	Control	0.35	0.06	0
30-60	Control	0.25	0.05	0
60-90	Control	0.24	0.12	0
0-30	Amm. nitrate	0.36	0.07	0
30-60	Amm. nitrate	0.35	0.07	0
60-90	Amm. nitrate	0.31	0.06	0
0-30	Urea	0.26	0.05	0
30-60	Urea	0.34	0.05	0
60-90	Urea	0.31	0.06	0
7 d after 40 kg N ha^{-1}				
0-30	Control	0.48	0.08	0
30-60	Control	0.51	0.11	0
60-90	Control	0.53	1.17	0
0-30	Amm. nitrate	1.67	1.63	0
30-60	Amm. nitrate	1.23	1.21	0
60-90	Amm. nitrate	2.05	2.50	0
0-30	Urea	0.70	0.12	2.36
30-60	Urea	0.80	0.13	4.01
60-90	Urea	1.00	0.19	7.42
7 d after 60 kg N ha^{-1}				
0-30	Control	0.45	0.20	0
30-60	Control	0.46	0.13	0
60-90	Control	0.43	0.17	0
0-30	Amm. nitrate	2.46	2.70	0
30-60	Amm. nitrate	1.84	0.82	0
60-90	Amm. nitrate	1.44	1.54	0
0-30	Urea	1.62	0.29	2.88
30-60	Urea	0.96	0.17	1.19
60-90	Urea	1.25	0.24	1.43
