# Supplementary material for: Seed Yield and Nitrogen Efficiency in Oilseed Rape After Ammonium Nitrate or Urea Fertilization
Source: Front Plant Sci. 2021 Jan 27;11:608785. doi: 10.3389/fpls.2020.608785 (PMC7874180; doi:10.3389/fpls.2020.608785)
Supplement: Supplementary Table 3 — Translocation rates of total N, amino acids, N forms and cytokinins in the xylem sap of 15 winter oilseed rape genotypes at the developmental stages BBCH57, BBCH65, and BBCH75 in the experimental years 2012/13 and 2013/14. [file Data_Sheet_6.PDF]

S3 Table. Translocation rates of total N, amino acids, N forms and cytokinins in the xylem sap of 15 winter oilseed rape genotypes at the developmental stages BBCH57, BBCH65 and BBCH75 in the experimental years 2012/13 and 2013/14. Values show means ±SD (n=4). Translocation rates of total N, amino acids and N forms are shown in µmol h^{-1} $plant^{-1}$, cytokinins are shown in pmol h^{-1} $plant^{-1}$. Asterisks show significant differences among respective compounds under ammonium nitrate vs. urea treatment according to unpaired t-test at p<0.05. Different upper/lower case letters show significant differences among the genotypes under ammonium nitrate/urea treatment according to Tukey´s test or Tukey´s test on ranks at p<0.05. ANOVA or ANOVA on ranks results *, **, *** show significant differences or interactions at p<0.05, p<0.01, p<0.001, respectively; G=genotype, T=treatment, ns=non-significant. n.d.=not detected. AN=Ammonium nitrate.

Genotype	BBCH57							BBCH65							BBCH75					
	AN	Urea		AN	Urea	AN	Urea	AN	Urea		AN	Urea	AN	Urea	AN	Urea	AN	Urea	AN	Urea
	Total N	Total N						Total N	Total N						Total N	Total N				
2012/13																				
PBC007	39.4 ±8.5	34.5 ±5.3						36.7 ±9.2	37.7 ±11.6						13.6 ±9.4	14.8 ±7.1				
PBC015	47.1 ±8.3	33.5 ±7.5						24.4 ±13.2	32.2 ±15.0						14.0 ±7.5	12.5 ±6.0				
Alpaga	43.6 ±9.7	36.4 ±12.2						16.8 ±4.5	19.3 ±10.8						7.8 ±4.4	10.0 ±7.9				
11091433	50.6 ±24.3	37.1 ±7.8						29.1 ±21.1	29.4 ±14.1						18.1 ±13.2	19.9 ±18.4				
12091707	48.0 ±6.5	33.6 ±15.2						24.1 ±8.9	27.6 ±18.5						10.8 ±4.8	8.0 ±3.8				
BCSNE001	50.1 ±7.0	59.6 ±20.0						25.0 ±7.7	27.1 ±8.4						11.2 ±3.8	10.5 ±5.9				
BCSNE002	44.2 ±11.4	42.8 ±10.8						31.4 ±8.8	15.7 ±12.3						15.8 ±7.0	8.7 ±8.4				
DSV-01	42.7 ±9.4	28.3 ±11.8						28.0 ±11.1	33.3 ±23.0						13.7 ±5.8	13.9 ±9.1				
DSV-02	40.4 ±7.4	37.2 ±6.6						32.9 ±23.3	21.4 ±10.9						13.9 ±9.4	9.1 ±5.9				
KWS_01	47.1 ±20.4	38.9 ±10.9						35.8 ±17.0	22.8 ±9.6						17.8 ±3.4	12.5 ±5.1				
KWS_02	50.7 ±16.3	29.6 ±16.3						33.6 ±18.9	13.6 ±5.6						11.6 ±7.2	8.7 ±8.0				
LG00-304E	53.1 ±22.0	52.6 ±26.4						23.5 ±14.7	32.6 ±26.7						15.5 ±6.1	13.6 ±13.9				
LG02-228D	45.2 ±7.4	24.3 ±10.1						31.2 ±21.4	22.8 ±6.6						11.4 ±8.8	6.1 ±3.6				
NPZ012	53.2 ±28.7	24.3 ±8.1	*					25.6 ±12.0	15.0 ±8.2						18.0 ±8.1	4.4 ±1.8				
NPZ208	45.8 ±9.9	38.1 ±11.8	*					31.5 ±10.7	20.8 ±2.6						8.9 ±4.8	8.2 ±5.4				
ANOVA	G ns, T ***, GxT ns							G ns, T *, GxT ns							G ns, T ns, GxT ns					
2013/14																				
PBC007	24.4 ±16.6	17.1 ±7.1	ab					6.8 ±1.2	5.7 ±2.3	AB					3.8 ±1.8	5.7 ±6.3				
PBC015	17.8 ±5.2	23.7 ±0.9	ab					4.6 ±2.5	7.9 ±2.1	AB					4.9 ±4.6	3.6 ±2.4				
PBC029	31.8 ±18.4	32 ±7	a					7.7 ±3.4	13 ±5.7	AB					2.7 ±1.1	5.2 ±2.8				
11091433	22.1 ±13.1	13.8 ±3	b					6.5 ±2.5	8.7 ±3.3	AB					3.8 ±1.4	3.2 ±1.5				
12091707	27 ±21.2	14.3 ±10.3	ab					6.4 ±2.7	8.7 ±3.6	AB					2 ±1.4	5.4 ±5.1				
BCSNE001	28.3 ±11.8	18.2 ±10.8	ab					6.8 ±4.7	8.7 ±0.7	AB					2.7 ±1.7	6.9 ±3.8				
BCSNE002	17 ±5.2	27.5 ±3.6	ab					4.7 ±0.6	7.3 ±1.6	AB					2.2 ±1.5	2.3 ±1.3				
DSV-01	20.5 ±6.4	19.5 ±4	ab					4.6 ±3.7	7.5 ±3.4	AB					2.2 ±0.9	2.4 ±0.2				
DSV-02	33.1 ±11.1	26.3 ±3.3	ab					11.2 ±2.4	6.3 ±4.3	A					4.1 ±2.4	3.1 ±1.2				
KWS_01	21.2 ±6.4	17.6 ±3.7	ab					5.1 ±2.4	12 ±4.4	* AB					1.6 ±0.6	6.6 ±8.1				
KWS_02	27 ±11.5	19.2 ±3.8	ab					3.1 ±1.6	4.9 ±3.4	B					3.4 ±1.3	1.5 ±1.1				
LG00-304E	18.8 ±5.5	21.4 ±10.1	ab					4.7 ±3	10.4 ±6.2	* AB					3.6 ±1.5	2.9 ±1.5				
LG02-228D	19.6 ±6.5	14.3 ±10	ab					7.2 ±2.2	9.6 ±3.1	AB					2.5 ±0.4	3.9 ±2.4				
NPZ012	21.6 ±15.7	23.5 ±12.8	ab					3.1 ±2.1	6.5 ±1.6	AB					2.4 ±1.3	4.1 ±2.7				
NPZ208	12.9 ±2.5	31.4 ±8.4	ab					8.5 ±4.4	9.9 ±5.5	AB					2.6 ±0.8	2.8 ±0.6				
ANOVA	G *, T *, GxT ns							G *, T ***, GxT ns							G ns, T ns, GxT ns					

S3 Table continued on next page. ►

S3 Table. Continued from previous page.

Genotype	BBCH57						BBCH65								BBCH75						
	AN	Urea	AN	Urea	AN	Urea	AN	Urea		AN	Urea		AN	Urea	AN	Urea		AN	Urea	AN	Urea
	Gln	Gln	Asp	Asp	Other AAs	Other AAs	Gln	Gln		Asp	Asp		Other AAs	Other AAs	Gln	Gln		Asp	Asp	Other AAs	Other AAs
2012/13																					
PBC007	6.3 ±1.7	6.4 ±1.0	4.6 ±0.4	4.2 ±1.0	2.6 ±0.8	2.6 ±0.9	4.3 ±0.9	4.6 ±0.6	ab	1.7 ±0.8	1.7 ±0.5		1.9 ±0.5	1.9 ±0.4	1.1 ±0.1	0.8 ±0.1	*	0.9 ±0.3	1.0 ±0.1	0.3 ±0.1	0.3 ±0.1
PBC015	7.5 ±2.6	7.5 ±1.1	3.3 ±0.4	4.1 ±0.6	3.2 ±1.0	2.6 ±0.6	4.1 ±1.3	4.3 ±0.9	ab	1.6 ±0.7	1.6 ±0.4		1.7 ±0.5	2 ±0.5	1.1 ±0.1	0.9 ±0.2		0.6 ±0.3	0.8 ±0.4	0.3 ±0.0	0.2 ±0.1
Alpaga	7.0 ±1.8	7.8 ±1.3	3.9 ±1.3	3.7 ±0.8	3.0 ±0.9	2.5 ±0.7	4.3 ±1.3	4.2 ±1.2	ab	2.1 ±0.3	2.1 ±0.9		1.4 ±0.3	1.7 ±0.1	0.9 ±0.2	1.1 ±0.4		0.9 ±0.3	1.0 ±0.6	0.2 ±0.0	0.3 ±0.1
11091433	7.5 ±1.6	7.2 ±1.5	3.9 ±1.0	3.7 ±1.2	1.9 ±0.1	2.1 ±0.3	5.6 ±2.6	4.4 ±1.2	ab	1.7 ±0.5	1.9 ±0.6		2.1 ±0.7	2.2 ±0.9	1.0 ±0.3	1.0 ±0.2		0.5 ±0.2	0.8 ±0.2	0.3 ±0.1	0.3 ±0.0
12091707	8.0 ±1.4	9.8 ±1.0	3.9 ±0.8	3.7 ±0.8	2.4 ±0.2	2.8 ±0.5	4.8 ±1.0	5.5 ±1.1	a	1.9 ±0.4	1.9 ±0.4		2.0 ±0.5	1.9 ±0.3	0.9 ±0.2	0.7 ±0.1		1.1 ±0.3	0.8 ±0.5	0.3 ±0.1	0.2 ±0.0
BCSNE001	6.7 ±0.8	6.2 ±0.5	4.4 ±1.7	4.3 ±1.2	2.5 ±0.6	3.3 ±0.6	4.3 ±0.7	4.2 ±0.5	ab	1.5 ±0.5	1.7 ±0.9		1.9 ±0.7	2.2 ±0.4	1.0 ±0.1	0.7 ±0.1		0.7 ±0.3	0.6 ±0.3	0.3 ±0.0	0.3 ±0.1
BCSNE002	6.7 ±1.2	6.7 ±1.0	3.5 ±1.0	3.8 ±1.1	2.9 ±0.5	3.1 ±0.9	5.5 ±2.5	3.8 ±0.7	ab	1.6 ±0.6	1.0 ±0.7		2.3 ±1.3	1.6 ±0.2	0.7 ±0.1	0.9 ±0.1		0.7 ±0.3	1.1 ±0.4	0.3 ±0.0	0.3 ±0.1
DSV-01	7.9 ±2.3	8.3 ±1.9	3.6 ±0.7	4.1 ±0.7	2.3 ±0.3	2.4 ±0.2	5.7 ±1.0	4.2 ±1.0	ab	1.8 ±0.8	1.2 ±0.4		2.4 ±0.5	1.7 ±0.2	0.9 ±0.2	1.2 ±0.2		1.2 ±0.2	0.9 ±0.4	0.3 ±0.1	0.3 ±0.1
DSV-02	9.1 ±0.8	7.2 ±2.9	4.0 ±1.1	3.6 ±0.7	2.6 ±0.7	3.0 ±0.6	4.9 ±0.9	4.1 ±0.5	ab	1.7 ±0.3	1.2 ±0.3		2.1 ±0.4	1.8 ±0.4	0.9 ±0.1	1.1 ±0.2		0.7 ±0.2	1.2 ±0.3	0.2 ±0.0	0.3 ±0.1
KWS_01	7.2 ±1.7	7.9 ±1.5	4.2 ±1.2	3.7 ±1.0	2.6 ±0.6	3.1 ±0.7	4.5 ±0.6	4.7 ±1.8	ab	1.5 ±0.4	1.7 ±0.5		2.1 ±0.7	2.2 ±1.0	1.2 ±0.5	1.0 ±0.2		1.1 ±0.1	1.2 ±0.4	0.4 ±0.1	0.3 ±0.1
KWS_02	6.9 ±2.3	7.6 ±2.0	4.9 ±1.0	4.2 ±1.0	2.2 ±0.3	2.5 ±0.5	6.4 ±3.4	4.9 ±0.8	ab	2.2 ±1.3	2.5 ±0.5		2.6 ±1.3	2.8 ±1.1	0.9 ±0.3	1.0 ±0.1		0.7 ±0.3	0.8 ±0.4	0.3 ±0.1	0.3 ±0.0
LG00-304E	7.6 ±1.7	6.9 ±1.7	4.8 ±0.6	3.5 ±1.2	2.4 ±0.9	2.7 ±0.7	4.9 ±1.0	4.5 ±0.9	ab	2.2 ±1.7	2.0 ±0.6		2.1 ±0.2	2.7 ±0.9	0.9 ±0.1	1.1 ±0.2		1.0 ±0.6	0.7 ±0.2	0.3 ±0.0	0.2 ±0.1
LG02-228D	7.3 ±1.8	6.0 ±1.3	3.7 ±0.6	3.3 ±1.4	2.1 ±0.4	2.5 ±0.7	4.4 ±0.5	3.7 ±0.9	ab	1.8 ±0.2	1.8 ±0.3		1.8 ±0.2	1.6 ±0.2	1.3 ±0.2	1.1 ±0.2	*	0.7 ±0.3	1.3 ±0.8	0.2 ±0.0	0.3 ±0.1
NPZ012	7.4 ±2.3	6.9 ±1.5	3.6 ±0.6	3.3 ±0.6	2.8 ±0.2	3.2 ±0.9	4.8 ±1.6	5.1 ±1.8	ab	1.1 ±0.7	1.2 ±0.2		1.8 ±0.3	2.0 ±0.3	1.1 ±0.2	0.7 ±0.1	*	0.6 ±0.1	0.5 ±0.3	0.4 ±0.1	0.3 ±0.1
NPZ208	8.6 ±0.6	7.0 ±1.9	3.5 ±0.6	4.7 ±1.5	1.9 ±0.5	2.5 ±0.6	4.3 ±1.0	2.2 ±0.5	b	1.5 ±0.5	1.7 ±0.9		2.1 ±0.1	1.3 ±0.4	1.1 ±0.2	0.8 ±0.1	*	1.2 ±0.4	1.2 ±0.3	0.3 ±0.1	0.3 ±0.1
ANOVA	G ns, T ns, GxT ns		G ns, T ns, GxT ns		G ns, T ns, GxT ns		G *, T ns, GxT ns			G ns, T ns, GxT ns			G ns, T ns, GxT ns		G ns, T ns, GxT *			G ns, T ns, GxT ns		G ns, T ns, GxT ns	
2013/14																					
PBC007	6.3 ±1.7	6.5 ±1.0	4.6 ±0.5	4.2 ±1.0	2.6 ±0.9	2.6 ±0.9	4.3 ±0.9	4.6 ±0.5		1.7 ±0.7	1.7 ±0.4		1.9 ±0.5	1.9 ±0.4	1.1 ±0.1	0.8 ±0.1		0.9 ±0.3	1.0 ±0.1	0.3 ±0.0	0.3 ±0.1
PBC015	7.5 ±2.6	7.5 ±1.1	3.3 ±0.4	4.1 ±0.6	3.2 ±1.0	2.6 ±0.6	4.1 ±1.3	4.3 ±0.8		1.7 ±0.7	1.6 ±0.4		1.7 ±0.5	2.0 ±0.5	1.1 ±0.1	0.9 ±0.2		0.6 ±0.3	0.8 ±0.4	0.3 ±0.0	0.2 ±0.1
PBC029	7.0 ±1.9	7.9 ±1.3	3.9 ±1.3	3.7 ±0.8	3.0 ±1.0	2.5 ±0.7	4.3 ±1.3	4.2 ±1.2		2.1 ±0.3	2.1 ±0.9		1.5 ±0.3	1.7 ±0.1	0.9 ±0.3	1.1 ±0.4		0.9 ±0.3	1.0 ±0.6	0.2 ±0.1	0.3 ±0.1
11091433	7.5 ±1.6	7.2 ±1.5	3.9 ±1.0	3.7 ±1.2	1.9 ±0.1	2.1 ±0.3	5.6 ±2.7	4.4 ±1.2		1.8 ±0.5	2.0 ±0.6		2.1 ±0.7	2.2 ±0.9	1.0 ±0.3	1.0 ±0.2		0.5 ±0.2	0.8 ±0.2	0.3 ±0.1	0.3 ±0.1
12091707	8.0 ±1.4	9.8 ±1.1	3.9 ±0.8	3.7 ±0.8	2.4 ±0.2	2.8 ±0.5	4.8 ±1.0	5.5 ±1.1		1.9 ±0.5	1.9 ±0.4		2.0 ±0.5	1.9 ±0.3	0.9 ±0.2	0.7 ±0.0		1.2 ±0.3	0.8 ±0.5	0.3 ±0.1	0.2 ±0.1
BCSNE001	6.7 ±0.9	6.2 ±0.5	4.4 ±1.7	4.3 ±1.2	2.5 ±0.5	3.3 ±0.6	4.3 ±0.6	4.2 ±0.5		1.5 ±0.6	1.7 ±0.9		1.9 ±0.7	2.2 ±0.4	1.0 ±0.1	0.7 ±0.1		0.8 ±0.3	0.7 ±0.2	0.3 ±0.0	0.3 ±0.1
BCSNE002	6.7 ±1.2	6.7 ±1.1	3.5 ±1.0	3.8 ±1.1	2.9 ±0.5	3.1 ±0.9	5.5 ±2.5	3.8 ±0.7		1.6 ±0.6	1.0 ±0.7		2.3 ±1.4	1.6 ±0.2	0.7 ±0.1	0.9 ±0.1		0.7 ±0.3	1.1 ±0.5	0.3 ±0.0	0.3 ±0.1
DSV-01	7.9 ±2.2	8.3 ±1.9	3.6 ±0.7	4.0 ±0.7	2.3 ±0.3	2.5 ±0.2	5.7 ±1.0	4.2 ±1.0		1.8 ±0.8	1.2 ±0.3		2.4 ±0.5	1.7 ±0.2	0.9 ±0.2	1.2 ±0.2		1.3 ±0.2	0.9 ±0.4	0.3 ±0.1	0.3 ±0.1
DSV-02	9.1 ±0.7	7.2 ±2.9	4.0 ±1.1	3.6 ±0.7	2.6 ±0.7	3.0 ±0.6	4.9 ±0.9	4.1 ±0.5		1.7 ±0.3	1.2 ±0.3	*	2.2 ±0.4	1.8 ±0.4	0.9 ±0.1	1.1 ±0.2		0.7 ±0.2	1.1 ±0.3	0.3 ±0.1	0.3 ±0.1
KWS_01	7.2 ±1.6	7.9 ±1.5	4.2 ±1.2	3.8 ±1.0	2.6 ±0.7	3.1 ±0.7	4.5 ±0.6	4.8 ±1.8		1.5 ±0.4	1.7 ±0.5		2.1 ±0.7	2.2 ±1.0	1.2 ±0.5	1.0 ±0.2		1.1 ±0.1	1.2 ±0.4	0.4 ±0.1	0.3 ±0.1
KWS_02	6.9 ±2.4	7.6 ±2.0	4.9 ±1.0	4.3 ±1.0	2.2 ±0.2	2.6 ±0.5	6.4 ±3.4	4.9 ±0.7		2.2 ±1.3	2.5 ±0.6		2.6 ±1.3	2.8 ±1.1	0.9 ±0.3	1.0 ±0.1		0.7 ±0.3	0.8 ±0.4	0.3 ±0.1	0.3 ±0.0
LG00-304E	7.7 ±1.7	6.9 ±1.7	4.8 ±0.5	3.5 ±1.2	2.4 ±0.9	2.7 ±0.7	4.9 ±1.0	4.5 ±0.9		2.2 ±1.7	2.1 ±0.6		2.1 ±0.2	2.7 ±0.9	1.0 ±0.1	1.1 ±0.2		1.0 ±0.6	0.7 ±0.2	0.3 ±0.0	0.3 ±0.1
LG02-228D	7.3 ±1.8	6.0 ±1.3	3.7 ±0.7	3.3 ±1.5	2.2 ±0.4	2.5 ±0.7	4.4 ±0.6	3.7 ±0.9		1.8 ±0.2	1.8 ±0.3		1.8 ±0.2	1.6 ±0.2	1.3 ±0.3	1.1 ±0.3		0.7 ±0.3	1.3 ±0.8	0.3 ±0.1	0.3 ±0.1
NPZ012	7.4 ±2.3	6.9 ±1.5	3.6 ±0.6	3.3 ±0.6	2.8 ±0.2	3.3 ±0.9	4.8 ±1.5	5.1 ±1.8		1.1 ±0.7	1.2 ±0.3		1.8 ±0.3	2.0 ±0.3	1.1 ±0.2	0.8 ±0.1		0.6 ±0.2	0.5 ±0.3	0.3 ±0.1	0.3 ±0.1
NPZ208	8.6 ±0.5	7.0 ±1.9	3.5 ±0.7	4.7 ±1.5	1.9 ±0.5	2.5 ±0.6	4.3 ±1.0	2.3 ±0.5		1.5 ±0.5	1.8 ±0.9		2.1 ±0.1	1.3 ±0.4	1.1 ±0.2	0.8 ±0.1		1.2 ±0.3	1.2 ±0.3	0.3 ±0.1	0.3 ±0.1
ANOVA	G ns, T **, GxT ns		G ns, T ***, GxT ns		G ns, T ns, GxT ns		G ns, T ns, GxT ns			G ns, T **, GxT ns			G ns, T ns, GxT ns		G ns, T ns, GxT ns			G ns, T ns, GxT ns		G ns, T *, GxT ns	

S3 Table continued on next page. ►

S3 Table. Continued from previous page.

Genotype	BBCH57									BBCH65							BBCH75					
	AN Nitrate	Urea Nitrate		AN Ammonium	Urea Ammonium		AN Urea	Urea Urea		AN Nitrate	Urea Nitrate		AN Ammonium	Urea Ammonium	AN Urea	Urea Urea	AN Nitrate	Urea Nitrate	AN Ammonium	Urea Ammonium	AN Urea	Urea Urea
2012/13																						
PBC007	13.1 ±2.4	11.3 ±3.6		0.2 ±0.0	0.2 ±0.0		n.d.	0.7 ±0.2	* ab	8.1 ±5.7	4.2 ±2.6	ab	0.1 ±0.0	0.1 ±0.1	n.d.	n.d.	5.1 ±1.1	3.1 ±1.0	n.d.	n.d.	n.d.	n.d.
PBC015	16.3 ±4.8	8.6 ±3.6	*	0.2 ±0.1	0.1 ±0.0		n.d.	0.5 ±0.2	* b	6.1 ±3.4	4.9 ±1	ab	0.1 ±0.0	0.2 ±0.2	n.d.	n.d.	2.7 ±1.0	2.2 ±0.7	n.d.	n.d.	n.d.	n.d.
Alpaga	14.2 ±3.7	8.0 ±2.2	*	0.2 ±0.1	0.2 ±0.1		n.d.	0.5 ±0.2	* ab	4.5 ±2.7	3.2 ±2.8	ab	0.2 ±0.1	0.1 ±0.2	n.d.	n.d.	5.1 ±2.3	5.8 ±5.4	n.d.	n.d.	n.d.	n.d.
11091433	11.2 ±0.2	7.2 ±1.5		0.3 ±0.1	0.2 ±0.1		n.d.	0.4 ±0.1	* ab	10.4 ±3.1	11.8 ±6.3	a	0.5 ±0.5	0.1 ±0.2	n.d.	n.d.	5.4 ±3.3	3.5 ±1.4	n.d.	n.d.	n.d.	n.d.
12091707	16.0 ±4.2	7.6 ±2.3	*	0.3 ±0.2	0.2 ±0.1		n.d.	0.5 ±0.1	* ab	9.5 ±1.6	9.7 ±6.9	ab	0.2 ±0.0	0.1 ±0.0	n.d.	n.d.	5.2 ±0.1	4.4 ±2.3	n.d.	n.d.	n.d.	n.d.
BCSNE001	13.6 ±4.3	7.4 ±1.9	*	0.2 ±0.1	0.1 ±0.1		n.d.	0.2 ±0.0	* b	8.9 ±5.1	8.3 ±0.6	ab	0.1 ±0.0	0.0 ±0.1	n.d.	n.d.	5.2 ±0.9	3.0 ±1.3	n.d.	n.d.	n.d.	n.d.
BCSNE002	14.8 ±5.1	8.8 ±4.2	*	0.3 ±0.2	0.2 ±0.1		n.d.	0.4 ±0.3	* ab	7.5 ±5.3	4.5 ±3.4	ab	0.2 ±0.1	0.2 ±0.1	n.d.	n.d.	2.1 ±1.5	7.1 ±4.4	n.d.	n.d.	n.d.	n.d.
DSV-01	11.7 ±7.5	12.8 ±5.1		0.4 ±0.2	0.3 ±0.1		n.d.	0.6 ±0.3	* a	3.3 ±2.1	4.4 ±2.3	ab	0.2 ±0.0	0.1 ±0.0	n.d.	n.d.	1.9 ±1.0	3.2 ±2.6	n.d.	n.d.	n.d.	n.d.
DSV-02	12.3 ±3.9	8.6 ±2.6		0.2 ±0.0	0.2 ±0.1		n.d.	0.4 ±0.2	* ab	6.9 ±3.4	5.6 ±2.5	ab	0.3 ±0.1	0.3 ±0.2	n.d.	n.d.	4.0 ±2.9	3.2 ±2.5	n.d.	n.d.	n.d.	n.d.
KWS_01	12.6 ±2.3	5.9 ±3.4	*	0.3 ±0.1	0.2 ±0.0		n.d.	0.3 ±0.0	* ab	5.7 ±3.5	4.5 ±2.7	ab	0.2 ±0.1	0.3 ±0.2	n.d.	n.d.	5.6 ±3.0	3.2 ±3.0	n.d.	n.d.	n.d.	n.d.
KWS_02	14.6 ±3.0	8.0 ±0.5	*	0.3 ±0.1	0.1 ±0.0		n.d.	0.6 ±0.1	* b	12.9 ±7.5	4.6 ±2.1	* ab	0.3 ±0.2	0.1 ±0.1	n.d.	n.d.	3.4 ±2.2	4.0 ±1.6	n.d.	n.d.	n.d.	n.d.
LG00-304E	13.0 ±5.6	8.7 ±3.0		0.2 ±0.1	0.2 ±0.1		n.d.	0.6 ±0.2	* ab	11.6 ±7.0	3.8 ±2.1	* ab	0.1 ±0.0	0.2 ±0.1	n.d.	n.d.	5.6 ±1.3	3.5 ±1.8	n.d.	n.d.	n.d.	n.d.
LG02-228D	13.0 ±4.5	5.7 ±2.2	*	0.2 ±0.1	0.2 ±0.1		n.d.	0.3 ±0.1	* ab	6.5 ±2.8	1.3 ±1.2	b	0.1 ±0.0	0.2 ±0.0	n.d.	n.d.	3.3 ±2.6	2.8 ±0.5	n.d.	n.d.	n.d.	n.d.
NPZ012	11.2 ±2.8	4.5 ±2.0	*	0.2 ±0.1	0.1 ±0.0		n.d.	0.2 ±0.1	* b	9.1 ±6.7	6.6 ±2.9	ab	0.4 ±0.4	0.3 ±0.3	n.d.	n.d.	2.7 ±1.4	5.7 ±3.8	n.d.	n.d.	n.d.	n.d.
NPZ208	14.5 ±6.1	4.1 ±1.1	*	0.3 ±0.1	0.1 ±0.0	*	n.d.	0.4 ±0.1	* b	7.1 ±2.3	2.9 ±2.6	ab	0.2 ±0.1	0.0 ±0.1	n.d.	n.d.	4.4 ±3.2	2.2 ±0.7	n.d.	n.d.	n.d.	n.d.
ANOVA	G ns, T ***, GxT ns			G ns, T ***, GxT ns			G *, T ***, GxT ns			G *, T **, GxT ns			G ns, T *, GxT ns				G ns, T ns, GxT ns					
2013/14																						
PBC007	7.1 ±4.4	5.2 ±2.0		0.3 ±0.1	0.1 ±0.1		n.d.	0.9 ±0.1	*	0.9 ±0.4	1.0 ±0.3		n.d.	n.d.	n.d.	n.d.	0.3 ±0.2	0.4 ±0.2	n.d.	n.d.	n.d.	n.d.
PBC015	10.2 ±1.8	5.6 ±0.8	*	0.3 ±0.2	0.4 ±0.4		n.d.	1.0 ±0.3	*	0.7 ±0.3	1.1 ±0.7		n.d.	n.d.	n.d.	n.d.	0.3 ±0.2	0.6 ±0.4	n.d.	n.d.	n.d.	n.d.
PBC029	9.9 ±5.2	6.0 ±2.0		0.5 ±0.1	0.4 ±0.2		n.d.	1.0 ±0.1	*	1.5 ±1.1	1.8 ±1.5		n.d.	n.d.	n.d.	n.d.	0.5 ±0.3	0.8 ±0.6	n.d.	n.d.	n.d.	n.d.
11091433	6.8 ±2.8	3.5 ±1.2		0.5 ±0.1	0.1 ±0.0		n.d.	0.9 ±0.5	*	0.5 ±0.4	0.8 ±0.5		n.d.	n.d.	n.d.	n.d.	0.5 ±0.4	0.6 ±0.3	n.d.	n.d.	n.d.	n.d.
12091707	11.3 ±4.2	3.9 ±1.9	*	0.4 ±0.1	0.3 ±0.0	*	n.d.	0.8 ±0.6	*	1.9 ±1.9	1.9 ±1.1		n.d.	n.d.	n.d.	n.d.	0.4 ±0.3	0.6 ±0.4	n.d.	n.d.	n.d.	n.d.
BCSNE001	8.6 ±4.3	3.9 ±2.5	*	0.3 ±0.2	0.1 ±0.0		n.d.	0.8 ±0.6	*	0.7 ±0.5	1.7 ±0.7		n.d.	n.d.	n.d.	n.d.	0.5 ±0.2	0.8 ±0.5	n.d.	n.d.	n.d.	n.d.
BCSNE002	9.3 ±2.1	6.1 ±0.9		0.4 ±0.1	0.1 ±0.0		n.d.	0.9 ±0.2	*	0.9 ±0.6	0.8 ±0.7		n.d.	n.d.	n.d.	n.d.	0.5 ±0.3	0.5 ±0.2	n.d.	n.d.	n.d.	n.d.
DSV-01	8.2 ±1.4	3.8 ±0.8	*	0.5 ±0.2	0.1 ±0.0		n.d.	0.8 ±0.4	*	1.1 ±0.6	2.7 ±2.2		n.d.	0.1 ±0.0	n.d.	n.d.	0.3 ±0.1	0.5 ±0.2	n.d.	n.d.	n.d.	n.d.
DSV-02	7.9 ±0.4	4.6 ±1.1		0.3 ±0.2	0.2 ±0.0		n.d.	0.8 ±0.1	*	1.7 ±0.9	1.3 ±1.1		n.d.	n.d.	n.d.	n.d.	0.5 ±0.3	0.3 ±0.3	n.d.	n.d.	n.d.	n.d.
KWS_01	8.0 ±1.7	3.9 ±1.2		0.3 ±0.1	0.1 ±0.0		n.d.	0.8 ±0.3	*	0.6 ±0.4	2.4 ±2.1		n.d.	n.d.	n.d.	n.d.	0.3 ±0.1	0.5 ±0.2	n.d.	n.d.	n.d.	n.d.
KWS_02	8.9 ±2.1	2.6 ±1.0	*	0.3 ±0.2	0.1 ±0.0		n.d.	0.7 ±0.2	*	0.5 ±0.3	1.3 ±1.1		n.d.	n.d.	n.d.	n.d.	0.3 ±0.3	0.5 ±0.3	n.d.	n.d.	n.d.	n.d.
LG00-304E	9.9 ±2.8	4.4 ±1.9	*	0.3 ±0.1	0.2 ±0.0		n.d.	0.7 ±0.5	*	0.3 ±0.3	1.0 ±0.5		n.d.	0.1 ±0.0	n.d.	n.d.	0.6 ±0.5	0.3 ±0.2	n.d.	n.d.	n.d.	n.d.
LG02-228D	7.4 ±1.8	3.4 ±1.1		0.7 ±0.2	0.1 ±0.1	*	n.d.	0.7 ±0.5	*	0.4 ±0.3	0.5 ±0.5		n.d.	n.d.	n.d.	n.d.	0.2 ±0.2	0.2 ±0.2	n.d.	n.d.	n.d.	n.d.
NPZ012	9.9 ±9.6	3.8 ±2.3	*	0.8 ±0.7	0.1 ±0.1	*	n.d.	0.9 ±0.6	*	1.3 ±0.8	1.2 ±0.4		n.d.	0.1 ±0.0	n.d.	n.d.	0.4 ±0.1	0.4 ±0.2	n.d.	n.d.	n.d.	n.d.
NPZ208	7.0 ±2.9	5.2 ±1.0		0.5 ±0.1	0.1 ±0.0	*	n.d.	1.0 ±0.1	*	0.8 ±0.7	0.8 ±0.4		n.d.	n.d.	n.d.	n.d.	0.1 ±0.1	0.2 ±0.1	n.d.	n.d.	n.d.	n.d.
ANOVA	G ns, T ***, GxT ns			G ns, T ***, GxT ns			G ns, T ***, GxT ns			G ns, T *, GxT ns			G ns, T ns, GxT ns				G ns, T ns, GxT ns					

S3 Table continued on next page. ►

S3 Table. Continued from previous page.

Genotype	BBCH57									BBCH65									BBCH75								
	AN	Urea		AN	Urea		AN	Urea		AN	Urea		AN	Urea		AN	Urea		AN	Urea		AN	Urea		AN	Urea	
	ZR	ZR		cZR	cZR		IPR	IPR		ZR	ZR		cZR	cZR		IPR	IPR		ZR	ZR		cZR	cZR		IPR	IPR	
2012/13																											
PBC007	51.6 ±8.1	35.8 ±6.8	AB	6.9 ±0.9	7.0 ±1.6	AB	11.2 ±4.8	8.4 ±2.6	AB	21.9 ±2.3	67.0 ±30.5		1.3 ±0.6	2.2 ±1.0		1.3 ±0.9	3.8 ±1.4	B	59.1 ±19.3	76.2 ±11.0	A a	2.0 ±0.3	2.3 ±0.2		7.2 ±3.2	15.7 ±6.5	*
PBC015	30.0 ±5.2	17.4 ±8.6	AB	7.4 ±2.0	4.9 ±1.5	AB	3.7 ±1.7	3.1 ±1.6	B	45.3 ±18.0	27.4 ±20.6		2.7 ±0.7	2.2 ±0.4		4.4 ±2.3	2.2 ±2.3	AB	20.4 ±9.5	38.4 ±23.6	AD ad	1.0 ±0.5	1.0 ±0.4		2.2 ±1.0	5.2 ±4.1	
Alpaga	61.8 ±13.8	41.3 ±21.7	AB	11.5 ±0.3	10 ±4.3	A	12.7 ±4.3	8.3 ±4.1	AB	124.4 ±13.0	96.9 ±51.0		3.0 ±1.1	2.9 ±1.8		11.1 ±7.5	5.1 ±4.1	AB	21.8 ±7.2	61.3 ±18.9	* AD ab	1.5 ±0.3	2.0 ±0.3		2.5 ±0.8	8.4 ±1.7	
11091433	29.7 ±3.6	20.2 ±2.5	AB	5.8 ±1.1	5.0 ±0.8	AB	3.8 ±1.6	4.3 ±1.3	B	126.3 ±49.1	81.0 ±57.0		6.4 ±3.0	7.4 ±6.5		23.7 ±14.2	16 ±9.9	AB	29.3 ±20.4	47.9 ±18.0	AD ad	2.3 ±2.0	1.9 ±0.3		4.3 ±4.3	6.6 ±3.5	
12091707	57.7 ±32.0	40.8 ±10.1	AB	9.8 ±5.3	8.7 ±2.5	AB	10.6 ±4.7	8.6 ±1.4	AB	118.1 ±95.5	127.4 ±39.1		4.0 ±3.1	3.8 ±1.4		10.9 ±10.5	8.9 ±4.3	AB	31.4 ±7.8	25.1 ±12.7	AD cd	2.1 ±0.7	1.2 ±0.3		8.2 ±4.9	5.7 ±2.9	
BCSNE001	44.0 ±13.4	32.2 ±12.2	AB	7.6 ±2.4	9.0 ±2.5	AB	6.8 ±1.4	6.7 ±2.9	AB	60.0 ±28.2	74.8 ±25.8		3.1 ±2.0	6.3 ±5.3	*	5.2 ±3.5	22.5 ±31.6	AB	48.8 ±32.5	24.7 ±17.1	AC cd	1.8 ±0.7	1.8 ±0.7		10.1 ±8.6	4.3 ±3.7	
BCSNE002	33.6 ±13.3	26.9 ±9.1	AB	7.4 ±2.6	7.8 ±2.4	AB	11.1 ±3.6	8.6 ±3.3	AB	84.6 ±38.0	73.7 ±52.8		3.7 ±1.5	2.9 ±1.0		8.3 ±3.4	8.2 ±5.8	AB	7.4 ±6.2	22.2 ±12.9	D cd	1.1 ±0.3	1.6 ±0.9		1.8 ±1.3	2.6 ±2.2	
DSV-01	58.1 ±12.1	49.3 ±7.8	AB	8.2 ±0.9	9.0 ±2.3	AB	7.6 ±1.7	7.8 ±0.8	AB	124.0 ±27.6	82.0 ±61.2		4.8 ±1.8	3.7 ±1.1		10.1 ±3.4	4.2 ±3.8	AB	9.1 ±2.1	46.6 ±3.6	* CD ad	1.0 ±0.5	2.5 ±0.8		2.1 ±1.6	9.8 ±1.8	*
DSV-02	43.4 ±11.1	55.3 ±13.0	AB	10.3 ±1.0	11.9 ±3.5	A	8.0 ±2.4	7.9 ±2.6	AB	153.5 ±67.6	118.8 ±72.4		2.8 ±2.2	4.4 ±3.1		13.2 ±9.7	17.3 ±19.9	AB	22.1 ±17.3	33.7 ±15.9	AD ad	2.7 ±1.2	3.0 ±2.4		8.0 ±9.9	6.5 ±4.9	
KWS_01	47.3 ±18.3	26.5 ±11.7	AB	10.3 ±2.6	7.8 ±3.2	AB	8.4 ±4.2	4.7 ±2.6	AB	46.1 ±30.0	53.1 ±22.7		3.4 ±1.6	3.0 ±1.2		3.0 ±1.7	3.9 ±0.8	AB	16.2 ±10.6	12.7 ±7.3	BD bd	1.9 ±1.0	1.4 ±0.7		3.1 ±2.9	1.6 ±0.9	
KWS_02	58.7 ±14.5	51.3 ±33.8	AB	8.6 ±3.3	10.1 ±6.3	AB	16.3 ±6.9	11.5 ±7.3	A	79.4 ±13.0	121.1 ±31.1		3.8 ±1.2	3.8 ±1.1		6.0 ±2.3	7.8 ±1.9	AB	44.0 ±20.8	45.4 ±38.3	AD ad	1.9 ±0.9	2.4 ±1.2		5.7 ±3.0	3.1 ±2.3	
LG00-304E	84.7 ±49.1	71.4 ±33.9	A	6.2 ±2.6	6.4 ±0.5	AB	13.4 ±6.5	12.6 ±5.9	AB	250.6 ±165.8	121.2 ±40.2	*	4.7 ±1.1	2.3 ±0.8		18.4 ±11.4	8.3 ±4.9	AB	22.6 ±12.2	56.6 ±17.9	* AD ac	1.6 ±0.4	1.2 ±0.8		6.0 ±5.9	4.3 ±1.8	
LG02-228D	58.4 ±22.4	33.6 ±19.3	AB	7.6 ±1.6	5.1 ±2.6	AB	15.3 ±6.7	6.7 ±4.5	A	170.9 ±50.6	33.9 ±10.9	*	4.2 ±1.6	1.4 ±0.6		19.0 ±13.4	2.7 ±1.2	* AB	54.0 ±15.0	64.1 ±29.3	AB ab	1.8 ±0.3	2.5 ±2.0		11.2 ±5.1	14.6 ±10.4	
NPZ012	51.7 ±8.5	36.6 ±17.8	AB	10.5 ±5.1	8.0 ±5.1	AB	8.9 ±1.6	7.0 ±3.4	AB	51.2 ±14.6	85.2 ±9.4		3.0 ±2.3	2.8 ±0.5		5.9 ±2.2	9.4 ±2.8	AB	13.4 ±8.8	9.4 ±7.9	BD d	1.2 ±0.1	1.7 ±1.3		2.6 ±2.1	2.2 ±1.9	
NPZ208	36.5 ±18.4	21.0 ±7.0	B	3.9 ±0.8	4.3 ±1.0	B	8.5 ±4.7	5.0 ±1.5	AB	201.2 ±122	109.0 ±35.0	*	4.6 ±1.7	3.2 ±1.8		28.5 ±16.6	16.8 ±6.4	A	19.8 ±8.1	29.1 ±12.1	AD bd	1.3 ±0.6	1.3 ±0.3		4.7 ±2.2	8.5 ±3.9	
ANOVA	G ***, T ***, GxT ns			G ***, T ns, GxT ns			G ***, T **, GxT ns			G ns, T ns, GxT ns			G ns, T ns, GxT ns			G ***, T ns, GxT ns			G ***, T ***, GxT ns			G ns, T ns, GxT ns			G ns, T ns, GxT ns		
2013/14																											
PBC007	24.6 ±17.5	25.9 ±8.9	AB	8.3 ±5.4	7.3 ±3.1	AB	4.3 ±2.9	4.8 ±1.1	AB	30.9 ±16.7	56.9 ±34.8		2.7 ±1.0	3.2 ±0.9	AB	5.0 ±3.3	10.4 ±7.0	AB ab	8.3 ±4.0	13.4 ±10.7	AB ab	1.4 ±0.1	2.2 ±2.2		1.6 ±0.7	2.5 ±2.4	
PBC015	28.2 ±9.9	11.8 ±3.5	AB	9.1 ±3.6	6.8 ±0.5	AB	3.5 ±2.7	1.7 ±0.6	AB	14.8 ±4.6	44.1 ±32.1		2.0 ±0.3	3.8 ±1.2	AB	2.7 ±0.4	5.4 ±2.9	BC ab	1.7 ±1.2	4.5 ±3.9	B b	1.9 ±2.0	1.0 ±0.5		0.8 ±0.8	1.5 ±1.9	
PBC029	36.5 ±17.3	25.8 ±7.1	AB	11.5 ±4.4	14.0 ±10.2	AB	6.1 ±3.1	7.8 ±4.4	AB	63.5 ±61.7	86.6 ±37.1		3.8 ±1.5	6.9 ±2.4	* AB	8.2 ±2.3	24.5 ±13.4	* A a	15.5 ±2.3	30.0 ±9.6	* A a	1.7 ±0.8	2.2 ±0.8		5.8 ±3.2	5.2 ±2.8	
11091433	11.4 ±5.1	20.8 ±14.0	B	4.0 ±1.4	6.1 ±2.1	B	1.4 ±0.5	2.6 ±1.2	B	20.9 ±11.6	29.8 ±11.8		2.9 ±1.5	3.5 ±1.6	AB	4.0 ±2.4	4.2 ±2.2	AC ab	9.1 ±5.0	16.2 ±8.4	AB ab	1.6 ±0.6	1.6 ±0.5		1.0 ±0.5	2.8 ±1.5	
12091707	41 ±14.5	25.4 ±14.7	AB	11.4 ±2.2	8.4 ±2.8	AB	5.5 ±1.3	6.1 ±3.0	AB	22.3 ±9.9	35.9 ±18.3		3.3 ±1.4	4.4 ±1.6	AB	4.2 ±1.6	9.2 ±4.9	AC ab	10.5 ±4.8	16.2 ±3.5	AB ab	2.9 ±3.0	1.7 ±0.5		3.0 ±1.9	3.6 ±1.7	
BCSNE001	29.5 ±19.2	23.5 ±19.0	AB	8.5 ±5.0	10.4 ±4.8	AB	3.5 ±2.5	6.0 ±5.4	AB	36.9 ±12.0	50.9 ±15.8		4.5 ±0.8	5.0 ±0.9	AB	5.7 ±2.2	10.2 ±3.0	AC ab	6.6 ±3.6	17.2 ±5.7	* AB ab	1.1 ±0.2	2.8 ±2.1		2.0 ±1.2	2.9 ±1.0	
BCSNE002	27.1 ±10	27.8 ±11.8	AB	9.3 ±3.4	10.7 ±3.1	AB	6.5 ±5.7	7.3 ±4.8	AB	10.5 ±6.4	23.1 ±12.4		1.9 ±0.8	3.6 ±1.1	AB	0.9 ±0.4	2.8 ±0.5	C b	4.1 ±2.5	4.4 ±1.6	AB b	0.8 ±0.2	1.6 ±1.0		0.9 ±0.3	2.3 ±2.0	
DSV-01	32.5 ±9.8	27.9 ±3.2	AB	8.8 ±3.0	8.3 ±0.7	AB	4.0 ±1.3	4.7 ±1.6	AB	27.5 ±15.2	64.7 ±23.1	*	2.8 ±0.8	5.3 ±1.8	AB	3.4 ±1.4	6.0 ±1.0	AC ab	5.1 ±3.1	20.3 ±9.0	* AB ab	1.0 ±0.6	1.9 ±0.4		2.1 ±2.4	5.3 ±3.9	
DSV-02	42.1 ±21.2	23.5 ±3.8	* AB	17.2 ±8.0	10.0 ±1.6	* A	7.3 ±5.3	4.6 ±1.3	AB	38.0 ±9.5	57.9 ±25.1		6.2 ±1.4	5.3 ±2.7	A	6.9 ±2.3	9.0 ±5.2	AB ab	11.2 ±4.9	12.9 ±11.2	AB ab	1.6 ±0.5	1.7 ±0.4		2.2 ±1.7	3.0 ±3.5	
KWS_01	48.1 ±22.3	35.2 ±5.2	AB	13.7 ±3.9	11.5 ±0.6	AB	4.7 ±0.9	5.5 ±0.6	AB	28.7 ±21.7	54.3 ±26.7		3.1 ±0.8	5.7 ±1.6	AB	3.6 ±1.1	8.4 ±4.1	AC ab	4.6 ±2.1	13.3 ±11.0	AB ab	1.1 ±0.6	2.0 ±1.0		1.2 ±1.0	2.8 ±2.0	
KWS_02	34.2 ±16.7	19.7 ±10.4	AB	10.2 ±4.4	7.2 ±3.3	AB	5.8 ±2.4	4.3 ±3.1	AB	19.0 ±10.0	30.2 ±22.8		1.8 ±0.7	3.4 ±1.5	B	2.9 ±2.1	7.7 ±8.7	AC ab	20.2 ±16.0	10.7 ±9.8	AB ab	1.8 ±0.7	1.5 ±0.5		2.4 ±1.9	1.5 ±1.6	
LG00-304E	57.6 ±16.3	37.2 ±10.0	* A	9.7 ±2.6	7.9 ±1.0	AB	8.6 ±1.0	5.6 ±2.2	A	49.6 ±41.2	72.5 ±22.7		3.4 ±1.7	4.4 ±1.7	AB	4.6 ±3.4	11.3 ±2.7	* AC ab	12.6 ±4.4	19.3 ±13.0	AB ab	1.3 ±0.2	1.6 ±0.8		1.9 ±1.2	3.2 ±2.8	
LG02-228D	16.6 ±6.6	19.1 ±2.5	AB	5.5 ±2.3	6.4 ±0.4	B	4.9 ±2.7	7.4 ±0.6	AB	25.0 ±14.0	31.2 ±5.8		2.5 ±0.8	2.8 ±0.9	AB	4.5 ±2.1	7.1 ±2.7	AC ab	15.3 ±10.0	12.1 ±8.0	AB ab	1.5 ±0.6	1.6 ±0.6		3.5 ±2.2	3.3 ±1.9	
NPZ012	23.6 ±12.5	26.3 ±7.6	AB	6.1 ±2.2	9.4 ±4.4	B	3.3 ±1.8	4.8 ±2.9	AB	29.8 ±24.4	30.8 ±29.6		5.8 ±7.4	3.4 ±1.1	AB	5.4 ±2.7	4.9 ±3.2	AC ab	5.8 ±0.6	8.8 ±5.3	AB ab	1.1 ±0.5	2.4 ±1.0		1.8 ±0.5	3.7 ±1.1	
NPZ208	22.9 ±10.1	21.2 ±5.5	AB	8.4 ±4.7	8.4 ±1.5	AB	4.0 ±1.8	5.6 ±2.9	AB	36.0 ±16.3	33.9 ±24.1		3.3 ±0.3	3.0 ±1.6	AB	6.5 ±2.5	8.0 ±7.5	AB ab	6.3 ±6.1	10.1 ±6.5	AB ab	0.9 ±0.6	1.3 ±0.8		2.3 ±2.0	3.3 ±2.2	
ANOVA	G ***, T **, GxT ns			G ***, T ns, GxT ns			G *, T ns, GxT ns			G ns, T ***, GxT ns			G **, T **, GxT ns			G ***, T ***, GxT ns			G ***, T ***, GxT ns			G ns, T *, GxT ns			G ns, T *, GxT ns		
